# Graphene Oxide Composites with Ultrahigh-Molecular-Weight Polyethylene for Innovative Prostheses for Arthroplasty

**DOI:** 10.1021/acsabm.5c01289

**Published:** 2025-12-15

**Authors:** Ahmed Subrati, Thyago Arruda Pacheco, Ítalo Azevedo Costa, Victor Carlos Mello, John Fredy Ricardo Marroquin, Mikołaj Kościński, Ludmila Alvim Gomes Pinho, Ariane Pandolfo Silveira, Jorlandio F. Felix, Marcilio Cunha-Filho, Marcio José Poças-Fonseca, Ricardo Bentes de Azevedo, Sonia Nair Báo, Rander Pereira Avelar, Leonardo Giordano Paterno, Sergio Moya, João Paulo Figueiró Longo

**Affiliations:** † 90216 Centro de Investigación Cooperativa en Biomateriales (CIC biomaGUNE), Donostia-San Sebastián, Guipúzcoa 20009, Spain; ‡ Institute of Biological Sciences, University of Brasilia, Brasilia 70910-900, Brazil; § Polymer and Nanomaterials Research Laboratory, Chemistry Institute, University of Brasilia, Brasilia, Distrito Federal 70910-900, Brazil; ∥ LabINS BSS 297, Institute of Physics, University of Brasília, Brasília 70910-900, Brazil; ⊥ Faculty of Physics and Astronomy, Adam Mickiewicz University, Uniwersytetu Poznańskiego 2, Poznań 61-614, Poland; # Department of Physics and Biophysics, Faculty of Food Science and Nutrition, Poznań University of Life Sciences, Wojska Polskiego 38/42, Poznań 60-637, Poland; ∇ Laboratory of Food, Drug and Cosmetics (LTMAC), School of Health Sciences, 28127 University of Brasília, Brasília, Distrito Federal 70910-900, Brazil; ○ CPMH Digital, Brasília, Distrito Federal 71200-260, Brazil

**Keywords:** dental prothesis, graphene oxide, UHMWPE, nanocomposite, arthroplasty, biocompatibility

## Abstract

Although ultrahigh-molecular-weight polyethylene (UHMWPE) is commonly used for prosthetic devices in arthroplasty, it is also prone to abrasion and wear. This wear can ultimately result in the release of particles that may trigger aseptic necrosis and activate the immune system. Graphene oxide (GO) incorporated into UHMWPE has been proven to be an effective strategy for improving the mechanical and biological properties of this commercial material. We aimed to incorporate graphene oxide into UHMWPE using an innovative method without toxic solvents and characterize this UHMWPE-GO prosthetic material in terms of its physicochemical and antimicrobial properties. The results showed that the UHMWPE-GO prosthesis, compared to traditional UHMWPE prostheses, exhibited improved mechanical properties, thermal stability, biocompatibility, and increased adhesion in human fibroblasts in culture conditions. Therefore, our work has produced a material for arthroplasty prostheses that offers improved performance and good biocompatibility.

## Graphical abstract

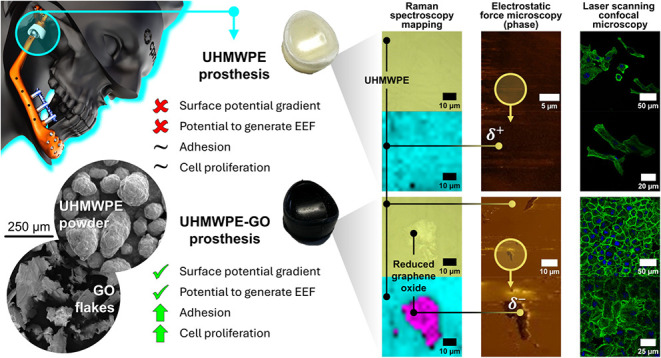

## Introduction

Joint arthroplasties have been prepared using ultrahigh-molecular-weight polyethylene (UHMWPE) since the 1960s due to its outstanding wear resistance and improved mechanical properties.^1^ This material is suitable for various types of joint prostheses, notably jaw and hip replacements.^2^ Despite UHMWPE’s efficient mechanical properties, its abrasion over time results in the generation of polyethylene fragments that can accumulate within the joint environment.^3^

The release of these particles from synthetic materials into the interstitial membrane of the bone joint activates fibroblasts and macrophages in the vicinity of the implant, leading to osteolysis that occurs under aseptic conditions.^4^ Moreover, these fragments can elicit foreign body responses, potentially causing aseptic necrosis in the affected area.^5^ In such cases, prosthetic replacement may become necessary, negatively impacting both the patient and the healthcare system.^1,5^ The increasing life expectancy of the population has also contributed to the need for increasing replacements each year.^1,6^

In this context, improvements in UHMWPE materials were developed to reduce these drawbacks. One important evolution was the development of highly cross-linked and stabilized UHMWPE that has significantly reduced wear rates and subsequent osteolysis in total joint arthroplasty; however, it has not eliminated the problem. As noted in Kandahari et al. (2016),^7^ long-term data on UHMWPE use are still lacking, and osteolysis remains a concern, especially in younger, more active patients. The ongoing need for medical intervention, as highlighted in the referred article, underscores the importance of continued research into novel material strategies to manage wear-related complications, even in the context of improved materials like highly cross-linked UHMWPE. Moreover, as demonstrated in Muratoglu et al. (2001)^8^ and Smith et al. (2025),^9^ increased cross-linking, while improving wear resistance, can come at the expense of fatigue resistance, potentially leading to other failure modes.

Thus, while UHMWPE offers improved mechanical properties for joint prostheses, its abrasion over time generates polyethylene fragments, leading to osteolysis (Kandahari et al., 2016),^7^ new technologies have been developed to overcome these drawbacks. In response to this challenge, researchers, in collaboration with industrial stakeholders, are exploring innovations to enhance the mechanical properties of these prostheses, thereby extending their lifespan^6^ and mitigating wear-related complications.

Among these initiatives, incorporating nanomaterials into prosthetic devices has emerged as an effective strategy to enhance the mechanical and physicochemical properties of these medical implants.^10^ Indeed, various types of nanomaterials, such as silica (SiO_2_) nanoparticles and fibers composed of *p*-phenylene terephthalamide, have been shown to improve the mechanical properties of UHMWPE.^11–13^ Furthermore, graphite nanoplatelets, carbon nanotubes, hydroxyapatite, nanosized calcium silicate (CaSiO_3_), and several other approaches have also been proposed in the literature.^14,15^

Particularly, graphene oxide (GO) emerges as a promising nanomaterial due to its biocompatibility and capacity to enhance the mechanical strength and durability of joint prostheses. For example, a previous study demonstrated that the incorporation of up to 1.0 wt % GO into UHMWPE increased the microhardness of prosthesis by approximately 22% and reduced the wear rate by about 55%.^16^ In fact, the incorporation of GO nanosheets into UHMWPE has been demonstrated to modulate mechanical properties and extend the lifespan of prostheses.^17–19^ Additionally, the electrical properties of reduced GO open interesting perspectives in the development of smart medical devices.^20^ In this scenario, a challenge remains to be overcome regarding the high hydrophilicity of GO, which makes its insertion into UHMWPE matrices difficult, and some processes use toxic solvents to better disperse the GO in the UHMWPE.^21–23^

Current methods for incorporating GO into UHMWPE use different raw materials, toxic solvents^24^ or several steps that increase the cost of producing the prosthesis.^25^ Furthermore, the same approaches are used in patented processes. Patent CN 107446221A, for example, on the one hand, uses dichlorobenzene, which can induce toxicological end points even after the compound has been removed. Patent US 2019 0211478, on the other hand, uses graphene paste, glass fiber, oil, and UHMWPE as a base, and these materials are processed by physically mixing them at high speed, ending with a technique for producing threads (gel-spun) and several steps that increase the cost of producing the prosthesis.

Thus, we aimed to develop an alternative method for incorporating GO into UHMWPE, focusing on industrial scalability and ease of production.^26^ The proposed innovative method consists of converting GO to reduced graphene oxide (RGO) in a solvent-free process easily inserted into UHMWPE, resulting in prostheses with better mechanical properties. This research involved a comprehensive strategy, encompassing material characterization and initial preclinical *in vitro* investigations. For the sake of simplicity, the fabricated materials presented in this work will be called prostheses. In clinical terms, these materials make up a part of the temporomandibular joint (TMJ) prosthesis, as demonstrated in Figure 1. TMJ prostheses are surgically implanted in patients to replace dysfunctional TMJ. The TMJ prosthesis consists of: Ti fossa plate, UHMWPE articular disc (condylar fossa component), Ti condylar sphere (condylar head), and Ti condylar plate.

**Figure 1:**
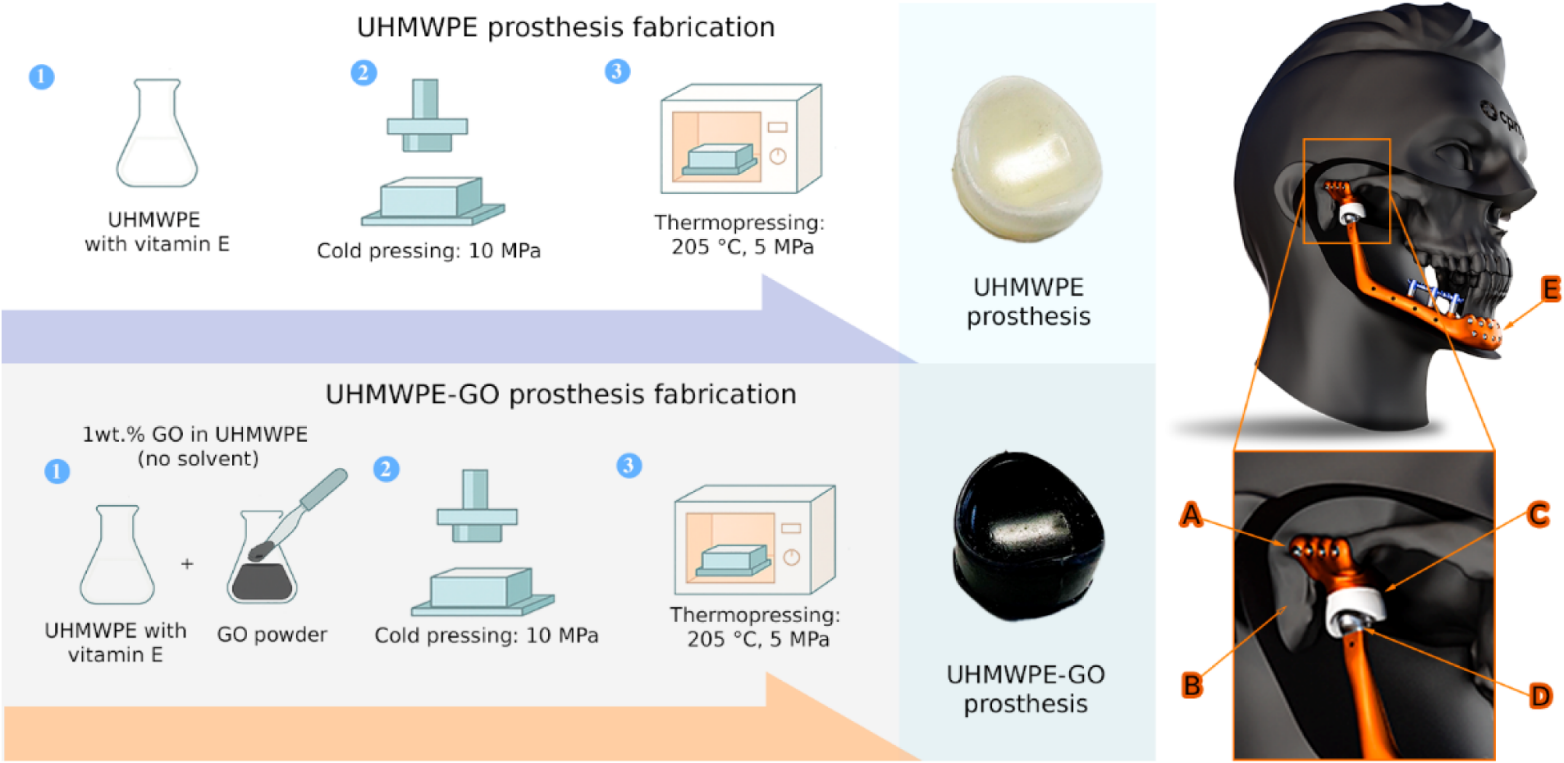
Scheme of prosthesis production. UHMWPE (top) and UHMWPE-GO (bottom). The model on the right depicts the CPMH Digital TMJ prosthesis. Labels A–E represent the Ti fossa plate, temporal fossa, UHMWPE articular disc (condylar fossa component), Ti condylar sphere (condylar head), and Ti condylar plate, respectively.

## Experimental Section

### Materials

#### Graphene and UHMWPE

Expandable graphite (flake size >300 μm), sulfuric acid 98%, potassium permanganate 99%, hydrochloric acid 37%, and hydrogen peroxide 30% were purchased from Sigma-Aldrich (San Luis, MO, USA) and used as received. UHMWPE enriched with vitamin E (GUR 1020-E natural, MW = 3.5 × 10^6^ g mol^–1^) was obtained from Celanese (São Paulo, Brazil). Deionized water was used in all steps of the GO preparation.

#### RAW 264.7 Cell Culture

The cells were cultured using Dulbecco’s Modified Eagle’s Medium (Gibco, Waltham, MA, USA), supplemented with 10% v/v fetal bovine serum (Gibco), in addition to 1% v/v penicillin solution and streptomycin at a concentration of 1000 U/mL (Invitrogen, Grand Island, NY, USA). The cells were kept in an oven at 5% CO_2_, 37 °C, and 95% humidity. RAW 264.7 was purchased from the American Type Culture Collection (ATCC), Sigma-Aldrich (Switzerland).

#### *Candida sp*. Culture

Cells were cultured for 48 h at 37 °C in Mueller–Hinton broth (MHB). MHB was purchased from Kasvi (Roseto degli Abruzzi, Teramo, Italy) and *Candida tropicalis*, *C. albicans*, *C. parapsilosis*, and *C. glabrata* strains were donated by Patricia Albuquerque (University of Brasilia, Brazil).

#### L132 Culture

Human fibroblasts (L132 cell line; ATCC CCL-5, Manassas, VA, USA) were cultured in Dulbecco’s Modified Eagle Medium—High Glucose (DMEM High; Gibco, Thermo Fisher Scientific, Waltham, MA, USA), supplemented with 10% v/v fetal bovine serum (FBS; Gibco), 1% v/v penicillin (10,000 U/mL)—streptomycin (10,000 μg/mL) solution (Invitrogen, Carlsbad, CA, USA). Cells were seeded at a density of 3 × 10^5^ cells per prosthesis and incubated at 37 °C in a humidified atmosphere containing 5% CO_2_ for 48 hours to allow adhesion to the surface.

### Synthesis of GO

GO was produced by exfoliation of graphite oxide, which was obtained according to the method described by Sun and Fugetsu.^27^ First, graphite was heated in a kitchen microwave, 10 min, and 800 W, to expand the graphite flakes. The heating was maintained until the graphite stopped sparking, generating a 10-fold increase in its volume. In the next step, 5 g of expanded graphite and 15 g of potassium permanganate were placed in a 1.0 L borosilicate beaker and manually mixed with a glass rod until a homogeneous powder was obtained. The beaker was then set within an ice bath, and 100 mL of sulfuric acid was added. The mixture was mechanically stirred at 500 rpm by using a Teflon helix until a liquid paste was formed. The ice bath was removed, and mechanical stirring continued until a foam-like cake was formed. Subsequently, 400 mL of deionized water was slowly added under mechanical stirring, transferred to a hot plate, and heated to 90 °C for 1 h under mechanical stirring. Finally, 10 mL of 30% hydrogen peroxide was added, and the mixture was left to cool at room temperature. The graphite oxide was purified by successive cycles of rinsing with deionized water, decantation, and discarding of the supernatant until it reached a pH of around 5.

After the graphite oxide synthesis, GO was obtained by exfoliation in deionized water with an ultrasonic disruptor (40 kHz, 150 W, pulsed mode, 2 h). The GO suspension was centrifuged to remove eventual aggregates and then dialyzed in water with a cellulose membrane with a 12,000 g/mol cutoff. The dialysis was run for 7 days, changing the water daily to remove ions and eventual byproducts of synthesis.

The GO suspension was freeze-dried in a Terroni benchtop lyophilizer, model LSE 3000B (São Carlos, Brazil). The GO was obtained as thin flakes, ground in a porcelain mortar, and sieved to obtain a particle size of less than 212 μm.

### Fabrication of Prostheses

UHMWPE commercially incorporated with vitamin E was manually mixed with the sieved GO powder (1 wt %). The mixture was transferred to a stainless-steel cast and kept cold-pressed under 10 MPa. The filled cast was then placed in an oven at 205 °C and 5 MPa pressure. Finally, the cast was removed from the oven and kept at room temperature under 10 MPa (patent number BR10202400456). Plain UHMWPE prostheses without GO were also produced by an identical procedure and used as a control (Figure 1).

### Characterization

#### Scanning Electron Microscopy (SEM)

SEM measurements were conducted on a JEOL SEM JSM-IT800HL (Tokyo, Japan) equipped with an energy-dispersive X-ray spectroscopy (EDS) system, ULTIM EXTREME SEM, with AZTEC software from OXFORD INSTRUMENTS PLC & SUBSIDIARIES (Abingdon, UK). Samples were supported on double-sided carbon tape (Ted Pella Inc., USA). Imaging of UHMWPE and GO powders, as well as prostheses of UHMWPE and UHMWPE-GO, was carried out at an accelerating voltage of 1.5 kV and a working distance of 4.3 mm. SEM data were processed using ImageJ. To conduct EDS analysis, the beam deceleration mode (gentle beam) was utilized at a working distance of 7.0 mm with an applied specimen bias of 2 kV (*E*_bias_) and an applied accelerating voltage or gun energy of 4 kV (*E*_gun_) in order to achieve a landing energy (*E*_landing_) of 2 kV, *E*_landing_ = *E*_gun_ – *E*_bias_.^28^

#### Transmission Electron Microscopy (TEM)

For high-resolution TEM (HR-TEM) studies, the GO was dispersed in ethanol. Before drop-casting on lacey carbon Cu grids (Ted Pella Inc., USA), the dispersion was subjected to soft sonication until it became homogeneous. The grid was left to dry under ambient conditions before the test. HR-TEM experiments were conducted on a JEOL JEM-2100F UHR electron microscope at 200 kV (Tokyo, Japan). HR-TEM data were processed by using ImageJ.

#### Contact Angle Measurement (CAM)

The wettability of the prostheses was investigated by a static water CAM system (DSA 100, Krüss, Germany) utilizing a sessile-drop method. A droplet volume of 2 μL was used for each measurement. The measurement time is 5 s. The results reported are averaged for two replicates.

#### X-ray Photoelectron Spectra (XPS)

X-ray photoelectron spectra were recorded by using the PHI XPS VersaProbe III energy spectrometer (Chanhassen, MN, USA) equipped with a monochromatic 1486.6 eV Al–Kα radiation source. A focused X-ray source with an X-ray beam size of 100 μm, 25 W power, and 15 kV e-beam energy was used. Charge neutralization was possible using a complementary dual-beam charge neutralization method. XPS data obtained from UHMWPE and GO powders, as well as prostheses made of UHMWPE and UHMWPE-GO, were processed by using XPSPEAK41.

#### X-ray Diffraction (XRD)

XRD measurements of UHMWPE and UHMWPE-GO prostheses as well as UHMWPE and GO powders were conducted on a PANalytical Empyrean X-ray diffractometer (Malvern, UK) featuring a copper tube, operating at 40 kV and 45 mA. Samples were scanned at room temperature within the angular range of 5–70° and 0.026 steps s^–1^. A Bragg–Brentano geometric optical arrangement was used. The Pearson VII function was applied to simulate and fit the XRD patterns. To estimate the percent crystallinity in prostheses, AMORPH^29^ was utilized within a 2θ window from 10° to 40°. The transverse crystallite size (*D*_110_) and longitudinal crystallite size (*D*_002_) were calculated by using the following equation:^30^

1 $$D_{\mathrm{hkl}}=\frac{0.\mathrm{89}\lambda }{\mathrm{fwhm cos}\theta }$$

where λ is the Cu Kα radiation wavelength of 1.54 Å, fwhm is the full width at half maximum of the diffraction signal of plane (hkl) in unit radians, and θ is the diffraction angle in unit radians.

#### Differential Scanning Calorimetry (DSC)

DSC measurements of the prostheses made of UHMWPE and UHMWPE-GO were conducted on a DSC-60 Shimadzu (Kyoto, Japan) to estimate the percent crystallinity (*X*_c_) by using the following equation:^31^

2 $$X_{c}=\frac{{\Delta H}_{m,i}}{{\Delta H}_{m,0}}\times 100$$

where Δ*H*_m,i_ and Δ*H*_m,0_ are the melting enthalpy of the sample and the fully crystalline polyethylene (293.0 J/g), respectively.^31^ Approximately 5 mg of samples were filled in aluminum pans for each run. The steps used for analysis were as follows: First, cooling from room temperature to −30 °C and holding for 3 min; second, heating from −30 °C to 230 °C and holding for 3 min; third, cooling from 230 °C to −30 °̊C and holding for 3 min; fourth, heating from −30 °̊C to 230 °̊C and holding for 3 min; fifth, cooling from 230 °̊C to −30 °̊C. All steps were performed at the same rate of 5 °̊C min^–1^ and under N_2_ at 200 mL min^–1^.

#### Raman Spectroscopy

Raman spectra were acquired by using an inVia Renishaw Raman microscopy system (Wotton-under-Edge, UK). A 532 nm laser line (1.29 mW power on stage) was used with a 50× L objective lens, 1800 lines mm^–1^ grating, extended, and a 1024 CCD array detector. The integration time was 10 s (5% of the maximum power), and each spectrum was a linear average of five distinct spectra collected from the remote regions of each sample. Spectra were analyzed by using OriginLab. All bands were simulated using Lorentzian functions apart from the D″ band, which was simulated using a Gaussian function following the method outlined in ref. 32. To estimate UHMWPE trans conformer content, the following equation was used:^33,34^

3 $$\mathrm{UHMWPE trans conformer content}\;\left(\%\right)=\frac{A_{1295}}{A_{1295}{+A}_{1305}}\times 100$$

where *A*_1295_ and *A*_1305_ are the integrated intensities of the bands found around 1295 cm^–1^ and 1305 cm^–1^, respectively.

Raman maps were acquired by using an inVia Renishaw Raman microscopy system. A 532 nm laser line (1.29 mW power on stage) was used with a 50× L objective lens, 600 lines mm^–1^ grating, static grating scan type, and a 1024 CCD array detector. Maps of 57 μm × 57 μm were acquired for UHMWPE-GO smooth and irregular surfaces with step sizes of 3 and 20 points along the *X* and *Y* directions. The integration time was 2 s (5% of the maximum power). The WiRE 4.4 software, attached to the instrument, was used to generate integrated intensity maps for UHMWPE and GO within Raman shift windows of 2790–2980 cm^–1^ and 900–1900 cm^–1^, respectively.

#### Thermogravimetric Analysis (TGA)

The thermal stability of UHMWPE and UHMWPE-GO prostheses, as well as precursor powders, was determined by TGA using a TA Instruments TGA Discovery (New Castle, DE, USA). Samples were filled in Pt pans and heated from room temperature to 800 °̊C at a rate of 5 °̊C min^–1^ under N_2_ at 20 mL min^–1^.

#### Mechanical Test

A universal testing machine, Shimadzu AG-IC 100kN (Kyoto, Japan), provided with a 10 mm diameter sphere plunger, was employed to record force vs displacement curves. Measurements were conducted at a 5 mm min^–1^ displacement speed at room temperature (25 °C). The samples were pressed until crushed to determine the maximum force of prostheses. The crushing test for the temporal fossa component was performed by an external company, CENIC Laboratory (Rua Oswaldo Denari, 165 - Munique, São Carlos - SP, 13568-600, Brazil). The company has expertise in conducting this type of test and is also accredited by the Brazilian health regulatory authorities (ANVISA) to perform such evaluations.

#### Atomic Force Microscope (AFM)

The sample surfaces of the prostheses were analyzed in air using a Nanosurf FlexAFM (Argóvia (AG), Switzerland), where a load was applied through the silicon tip (TAP 150 Al-G, Budget Sensors) featuring a force constant of 5 N m^–1^ and a resonance frequency within the range of 150 kHz. AFM data were analyzed by using Gwyddion software.

#### Electrostatic Force Microscopy (EFM)

EFM measurements were carried out using a Park NX7 Systems atomic force microscope operating in noncontact mode (NCM) with the lift mode function enabled. All measurements were performed in ambient air at room temperature. Probes of the MESP-V2 type (Bruker, USA) were used. These are antimony-doped n-type silicon cantilevers coated with a conductive Co–Cr alloy. The probes feature a nominal resonance frequency of approximately 75 kHz, a spring constant of 3 N m^–1^, and a tip radius of 35 nm, making them suitable for high-resolution electrostatic imaging. During EFM measurements, the lift height was set to 100 nm and a DC voltage of 10 V was applied to the samples. The samples were affixed to stainless steel AFM stubs using conductive silver paste and grounded to ensure stable electrostatic conditions. Data analysis was performed by using the Gwyddion software.

#### The RealTime-Glo Macrophage Viability Assay (RAW 264.7)

When cells reached a confluence of 2 × 10^4^ cells, they were plated into wells of a 24-well plate. Murine cells (RAW 264.7) were seeded on GO and UHMWPE powders dispersed in alcohol (0.6 mg/mL) or growing medium (1% GO in UHMWPE w/w). For the viability test, the Promega RealTime-Glo MT Cell Viability assay kit was used, and the luminescence of viable cells was evaluated by using Varioskan LUX (Thermo Scientific, Waltham, MA, USA). The assay procedure includes introducing NanoLuc luciferase and a cell-permeable prosubstrate into cultured cells.

#### *Candida species* Viability Assay

Prior to the experiments, GO and UHMWPE were exposed to 70% ethanol for 24 h for sterilization. In 96-well polystyrene plates, about 10^4^ yeasts per well were cultured at 37 °C in Mueller–Hinton broth (MHB, Kasvi (Roseto degli Abruzzi, Teramo, Italy)) in control conditions (only broth or 70% ethanol in broth), only with GO (0.6 mg/mL), only with UHMWPE (0.6 mg/mL), 24 mg/mL GO (1% w/w) + UHMWPE or 48 mg/mL GO (1% w/w) + UHMWPE. The BacTiter-Glo kit from Promega (Madison, USA) was employed to evaluate Candida spp. viability after 24 h growth. The amount of ATP, as an indicator of viable cells, was determined by luminescence reading over time (Varioskan LUX, Thermo Scientific, Singapore). For significant results in all *in vitro* tests, the statistical *t*-test and ONE-WAY ANOVA were used to make comparisons between the groups. A reliability value of 95% was considered, with *p* < 0.05 being statistically significant. The results were graphically represented using GraphPad software version 7.0.

#### Determination of *C. tropicalis* Colony Forming Units (CFU)

An isolated colony was cultured in MHB (Kasvi) at 37 °C for 24 h in an orbital shaker (150 rpm). The 10^4^ cells were spread onto Mueller–Hinton agar (Kasvi) plates containing different concentrations of GO or UHMWPE powders. Plates were then incubated at 37 °C for 48 h, and CFU were counted for each experimental condition. Additionally, about 10^4^ *C. tropicalis* yeasts were spread onto MHA plates where UHMWPE or UHMWPE-GO prostheses were placed in the center. Plates were then incubated at 37 °C for 48 h.

#### Plaque-Forming Unit (PFU)

*Candida tropicalis* culture was carried out following the same parameters as those of the other tests presented here. The protocol of Morais et al. 2020 ^35^ was followed for PFU with the dilutions and the challenge was only the concentration of 0.12 g/mL of UHMWPE with 1% (w/w) GO.

#### Powders Swab

0.4 g of each powder, GO and UHMWPE, was mixed and 10^4^ cells of *Candida tropicalis* was spread with a platinum loop over the powders placed on the surface of each plate, simultaneously spreading the powders and the *Candida tropicalis*.

#### Laser Scanning Microscopy

Human fibroblasts (L132 cell line) were cultured in a DMEM high medium, with a cell density of 300,000 cells per prosthesis. Following cell seeding, the prostheses were incubated for 48 h for surface adhesion. After incubation, adhered cells were fixed with 4% paraformaldehyde (Electron Microscopy Sciences, Hatfield, PA, USA) in phosphate-buffered saline (PBS; pH 7.4; Sigma-Aldrich, St. Louis, MO, USA) for 20 min at room temperature. Samples were then washed three times with PBS for 20 min each. Cell membranes were permeabilized with 0.1% Triton X-100 (Sigma-Aldrich) in PBS for 20 min. For cytoskeletal labeling, cells were incubated with Phalloidin conjugated to Alexa Fluor 488 (5 units per well; Thermo Fisher Scientific) for 20 min in the dark, followed by three 20 min PBS washes. Nuclear staining was performed using 100 μL of DAPI solution (500 nm; Thermo Fisher Scientific) for 5 min, followed by three additional PBS washes. Coverslips were mounted by using ProLong Gold Antifade Mountant (Thermo Fisher Scientific), and samples were imaged by using a TCS SP5 laser scanning confocal microscope (Leica Microsystems, Wetzlar, Germany).

## Results and Discussions

To answer the objective of our study, we have developed an alternative method for incorporating GO into UHMWPE, prioritizing industrial scalability and ease of production. Our innovative approach involves converting GO to reduced graphene oxide (RGO) in a solvent-free process, facilitating its insertion into UHMWPE to produce materials for prostheses with enhanced mechanical properties. This research employs a comprehensive strategy, encompassing thorough material characterization and initial preclinical *in vitro* investigations, to elucidate the physicochemical and antimicrobial properties of the resulting UHMWPE-GO composite, as these have been proven to be an effective strategy for improving the mechanical and biological properties of this innovative material.

### Morphological and Chemical Characterization

The morphologies of the micro- and nanostructures of UHMWPE and GO powders, the precursors for UHMWPE and UHMWPE-GO prostheses, are shown in Figure 2a–h. The UHMWPE beads (Figure 2a–b), with an average size of 87.6 ± 4.6 μm, exhibited an irregular, rounded shape. Their surfaces featured valleys densely populated with threads or tethers of UHMWPE. Such structures, known as fibrillar bundles, connect lamellar noduli that had an average size of 952 ± 31 nm.^30^

**Figure 2:**
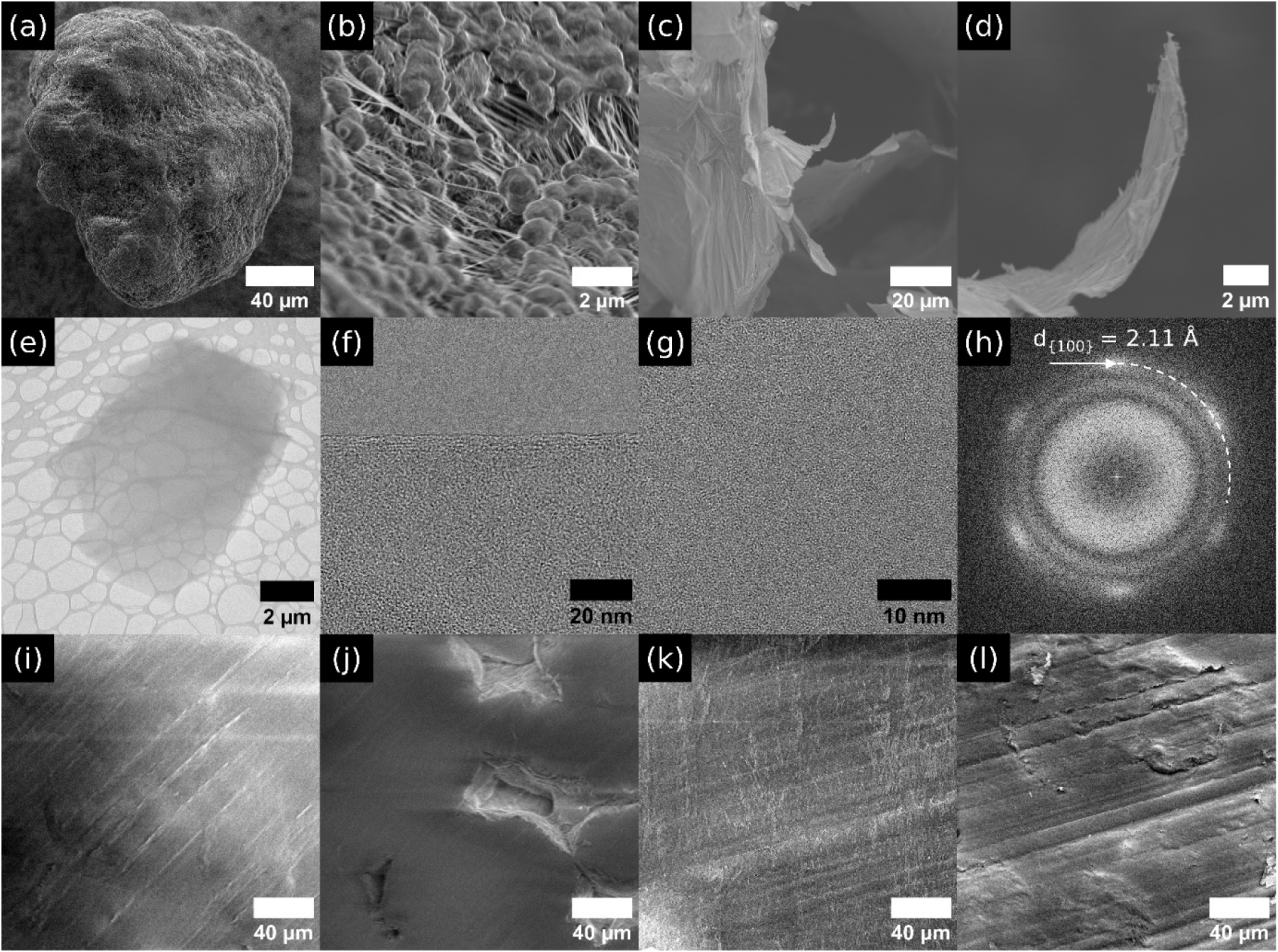
Electron microscopy of UHMWPE and GO powders and their prostheses showing morphological differences: (a) SEM image of UHMWPE bead, (b) close-up SEM image of the surface of the bead revealing microsized structure, (c) SEM image of GO thin flakes, (d) close-up SEM image of the GO flake, (e) low-resolution TEM image of a GO flake, (f) high-resolution TEM image of the edge of a thin flake, and (g) high-resolution TEM image of the bulk region of a thin flake exhibiting a discernible electron diffraction pattern of graphene (h). SEM images of smooth (i) and irregular (j) surfaces of the UHMWPE prosthesis. SEM images of smooth (k) and irregular (l) surfaces of the UHMWPE-GO prosthesis.

The GO powder was very flaky in nature and had a very thin and fluffy morphology (Figure 2c–e). Our results estimated the GO flake size by SEM with percentile values D10, D50, and D90 of 0.20, 0.38, and 1.19 μm, respectively (Figure S1). The high-resolution TEM images of GO (Figure 2f–g) revealed a thin segment of a flake that exhibited the intrinsic hexagonal array electron diffraction pattern (Figure 2h) of a Bernal graphene material,^32^ revealing an interlattice spacing for the {100} family of planes of 2.11 Å.^36^ GO powder exhibited an X-ray diffraction signal around 2θ of 11.0°, corresponding to the interlayer spacing of 8.03 Å (Figure S1). GO crystallite thickness was 11.1 nm (∼15 layers),^37^ confirming defective oxygen functionalities such as hydroxyls, epoxides, ethers, carbonyls, carboxyls, lactones, and lactols. These functional groups exhibited steric hindrance, expanding interlayer spaces in GO while preserving some sp^2^ domains’ percolation.^37^

We further investigated the surface morphologies of both UHMWPE and UHMWPE-GO prostheses using SEM (Figure 2i–l). Both materials exhibited a combination of smooth and irregular sections. The smooth sections in both prostheses displayed shallow perikymata and abrasion defects (Figure 2i,k). However, the irregular sections differed between them. UHMWPE prostheses were characterized by pits (Figure 2j), while UHMWPE-GO prostheses exhibited humps (Figure 2l),^38^ which was also corroborated by SEM/EDS elemental maps shown in Figures S2 and S4, respectively. Elemental analysis via SEM/EDS of fragments of both prostheses was carried out, as shown in Figure S2. The carbon-to-oxygen atomic ratio (C/O) was 98.0 and 40.3 for UHMWPE and UHMWPE-GO prostheses, respectively (Figure S3). UHMWPE and GO precursor powders were also analyzed via SEM/EDS (Figure S4), and C/O values of 624 and 2.9 were found, respectively (Figure S5). To enhance our understanding of the surface characteristics, we employed AFM to examine the prostheses, as shown in Figure S6. Interestingly, the average roughness of the UHMWPE prosthesis was measured at 37.14 nm, compared to 32.16 nm for the UHMWPE-GO prosthesis. These values represent the average roughness across various sections of the surfaces, highlighting the effect of incorporating GO sheets into the composite material. This incorporation reduced surface irregularities, which can otherwise harbor bacteria and facilitate their growth and colonization.^38^

Prostheses made of UHMWPE and UHMWPE-GO exhibit hydrophobicity; θ >90°, with the former having a contact angle of 93.50 ± 0.30° and the latter having a contact angle of 94.93 ± 0.98°, as shown in Figure S7. The estimated contact angle was slightly larger for UHMWPE-GO, which could be explained by the dispersibility issues of GO sheets within the prostheses or surface roughness factors. This finding suggested the successful incorporation of hydrophobic graphene sheets into the composite material. The hydrophobicity of GO was caused by the reduction of the oxygen content of the original GO powder due to the high temperature applied during thermopressing in the fabrication process of the UHMWPE-GO prosthesis. This was confirmed by Raman and XPS measurements, as shown below.

The high-resolution C 1s XPS deconvolution results of UHMWPE, GO, UHMWPE prosthesis, and UHMWPE-GO prosthesis are shown in Figure 3a–d and summarized in Table S1. Spectra were deconvoluted into one to six components: C=C (in graphitic domains), C–C (in hexagonal sp^3^ lattice or saturated carbons), C–O (in hydroxyl and epoxy groups), C=O (in carbonyl groups), COO (in carboxyl and lactone/lactol groups), and π-π* (graphitic transitions).^32,37,39^ The powder of UHMWPE had two distinct components, C–C and C–O, which were attributed to PE macromolecules and vitamin E, respectively. The GO powder had the above-mentioned components and exhibited the lowest carbon-to-oxygen atomic ratio of 2.4, which suggested a high oxidation extent due to the abundance of oxygen groups such as hydroxyl, epoxy, carbonyl, carboxyl, and lactone/lactol. The UHMWPE prosthesis depicted a single C–C component with an absent C–O component. This result could be explained by the plasticizing effect of vitamin E, wherein the plasticizing agent was finely dispersed and firmly latched within the PE macromolecules, providing enhanced consolidation,^40^ as illustrated in Figure S8. The UHMWPE-GO prosthesis C 1s spectrum revealed the presence of most innate UHMWPE and GO components with an anticipated substantial decrease of the C—C and C–O components with a predominance of the C–C component related to the thermopressing process. Worthy of mention is the possibility of overlap of the C–C components arising from UHMWPE and GO, as well as the C–O components originating from both GO and vitamin E.

**Figure 3:**
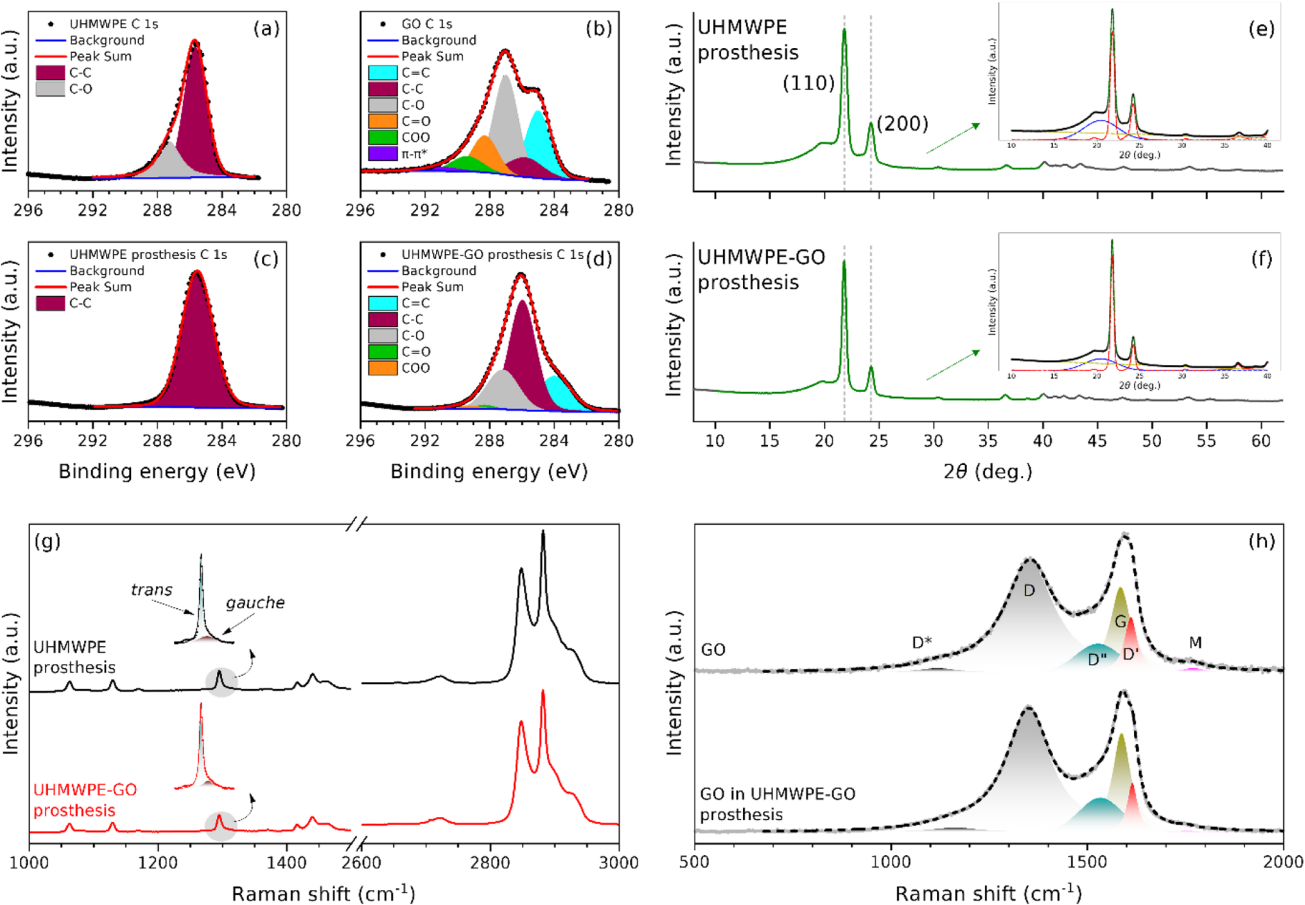
C 1s XP spectra of (a) UHMWPE powder, (b) GO powder, (c) UHMWPE prosthesis, and (d) UHMWPE-GO prosthesis. XRD of (e) UHMWPE prosthesis and (f) UHMWPE-GO prosthesis. The green part of the diffraction pattern was input into AMORPH^29^ for quantification of amorphous and crystalline phases. Insets show AMORPH^29^ analysis of the corresponding XRD patterns showing: a broad amorphous component in blue, crystalline component in red, linear background in yellow, model sum in green, and raw input diffraction data as black translucent circles. (g) Raman spectra of UHMWPE and UHMWPE-GO prosthesis with an inset showing the trans–gauche deconvolution. (h) Deconvolution of Raman spectra of GO powder as well as GO probed in the UHMWPE-GO prosthesis defect.

The XRD patterns of UHMWPE and UHMWPE-GO prostheses are shown in Figure 3e–f. Two intense diffraction peaks, namely (110) and (200), were identified, corresponding to the orthorhombic crystal structure of UHMWPE.^30,41^ From these two peaks, the lattice parameters a and b had been determined to be 7.33 and 4.90 Å, respectively. A peak around 2θ of 40.08° corresponded to the (011) plane, from which it was possible to estimate lattice parameter c to be 2.53 Å. No discernible variations were observed in terms of the lattice parameters. However, the AMORPH^29^ analysis, shown as insets in Figure 3e–f, had revealed an interesting difference. The amorphous content was 45.73 ± 0.68% in UHMWPE and 37.57 ± 0.51% in UHMWPE-GO. Despite the low amount of GO fed into the thermopressing process (1 wt % relative to UHMWPE) to produce the prostheses, the effect was drastic in terms of the crystallinity of the final prostheses UHMWPE-GO. GO certainly enhanced the crystallinity of the prostheses.

*D*_110_ and *D*_200_ values of the UHMWPE prostheses were estimated, using eq 1, to be 14.2 and 13.5 nm, respectively. The UHMWPE-GO prostheses’ *D*_110_ and *D*_200_ were 18.7 and 16.3 nm, respectively. The increase in transverse (preferential) and longitudinal crystallite sizes substantiated the AMORPH and DSC estimates of percent crystallinity, where the UHMWPE-GO prostheses were consistently found to be ca. 8% more crystalline than the UHMWPE prostheses. This finding informed about the ability of incorporated GO to favorably orient PE macromolecules in the UHMWPE-GO prostheses owing to strong adhesion between the nanofiller GO and the PE matrix.^42^ Another notable difference is the intensity ratio between the (110) and (200) planes, *I*_(110)_/*I*_(200)_, which was found to be 3.14 and 4.46 for the UHMWPE and UHMWPE-GO prostheses, respectively. This discernible difference indicated that GO influenced textural changes to facilitate the rearrangement of fibrils into fold crystals.^30^ Additional information could be deduced when comparing the values of *D*_110_ and *D*_200_ of the prostheses to those of the original UHMWPE powder (54.52% crystallinity—AMORPH determined as shown in Figure S9). *D*_110_ and *D*_200_ of the original UHMWPE powder were estimated to be 17.2 and 13.3 nm. On the one hand, the UHMWPE prosthesis fabrication did not affect the *D*_200_ but discernibly reduced the *D*_110_. On the other hand, the fabrication of the UHMWPE-GO prosthesis increased both *D*_110_ and *D*_200_. The *I*_(110)_/*I*_(200)_ of the UHMWPE powder was found to be 3.70, higher than that of the UHMWPE prosthesis and lower than that of the UHMWPE-GO prosthesis.

Figure S10 shows the DSC curves of prostheses made of UHMWPE and UHMWPE-GO, in which the melting peak was around 135 °̊C. Percent crystallinity (*X*_c_) was estimated using eq 2.^31^ Surprisingly, the estimated crystalline content by DSC corroborated the XRD data, as shown in Table 1. A consistent difference of 8% between both prostheses was maintained, with the UHMWPE prosthesis exhibiting the most amorphous content.

**Table 1:** Summary of Crystallinity (in Percentage) of the Prostheses as Determined by XRD and DSC

	Crystalline content (%)	
Prosthesis	XRD	DSC
UHMWPE	54.27	57.05
UHMWPE-GO	62.43	64.95

Figure 3g shows the Raman spectra of the surfaces of the UHMWPE and UHMWPE-GO prostheses. Both spectra exhibited strong bands characteristic of UHMWPE.^33^ It was interesting to note that the characteristic peaks of graphene, namely the D and G bands,^43^ were not evident in the spectrum of the UHMWPE-GO prosthesis but were strong when a defective region on the surface was probed, as shown in Figure S11. It seems as if, on the one hand, graphene layers were frozen and unable to breathe (Raman vibrations) in intact surfaces, and on the other hand, the layers can breathe in those microscale defective regions recurring on the surfaces of the UHMWPE-GO prosthesis. The most intense bands found around 2848 and 2881 cm^–1^ were attributed to the symmetric and asymmetric stretching vibration modes of CH_2_ groups, respectively.^33^ The bands found around 1295 and 1305 cm^–1^ were attributed to the CH_2_ twisting vibrations of CH_2_ groups adopting trans conformations (populating both crystalline and noncrystalline domains) and gauche conformations (solely making up the noncrystalline domains), respectively.^31^ Remarkably, the deconvolution of both bands, shown as insets in Figure 3g, allowed us to estimate the content of trans conformers: 59% and 78% trans-conforming PE macromolecules, respectively, made up the UHMWPE and UHMWPE-GO prostheses. The 19% increase in the more energetically stable conformation came with incorporating GO into the UHMWPE blend. In fact, an expected trait as fillers can increase the content of groups assuming the trans conformation.^33^

Analysis of Raman spectra of GO before and after incorporation into the UHMWPE matrix could inform about the structural changes brought to GO sheets and probable transformations caused by the interactions between GO and UHMWPE during the thermopressing process (Figure S11). Figure 3h presents the Raman spectra of GO before and after the thermopressing process. GO after thermopressing and shaping, was Raman-visible through microscale defects in the surface of the UHMWPE-GO prosthesis—see Raman maps in Figure S12. The spectra were deconvoluted into 6 bands: D*, D, D″, G, D′, and M. Table S2 summarizes the deconvolution results. The D* band is attributed to C–C and C=C stretching vibrations in polyene-like disordered graphitic structures.^44–46^ The D band is induced by disorder, activated by the intervalley scattering process, is caused by the emergence of defects, and is dependent on edge structure.^47^ The D″ band is ascribed to amorphous phases in graphene oxide and depends on crystallite size.^46,48^ Alternatively, out-of-plane deformations caused by mixed vibrational modes of sp^2^ carbons in proximity to defects originate in the D″ band.^49^ The G band is the graphitic band originating from the in-plane C=C stretching vibrational mode.^32^ The D′ band is induced by disorder, activated by the intravalley scattering process, is caused by the emergence of defects, and is not dependent on edge structure.^47^ The M band, sensitive to interlayer forces and typically hard to discern, is attributed to the out-of-plane layer-breathing vibration mode.^50^ Carbonyl/carboxyl/lactone stretching mode serves as another attribution for the M band.^49^ The peak integrated intensity ratio between the D band and the G band (*A*_D_/*A*_G_) is an excellent descriptor of defect density,^51^ from which one can estimate the crystallite width (*L*_a_).^37^ Interestingly, the peak integrated intensity ratio between the D band and the D′ band (*A*_D_/*A*_D’_) can help identify the nature of defects.^51^ GO had an *A*_D_/*A*_G_ of 3.23 (*L*_a_ of 6.0 nm), while GO in the UHMWPE-GO prosthesis had an *A*_D_/*A*_G_ of 3.12 (*L*_a_ of 6.2 nm). Reduction of GO, loss of some of the oxygen groups, was the main contributing factor to the decrease in the *A*_D_/*A*_G_. The *A*_D_/*A*_D’_ for GO was 8.02, whereas it was 11.34 for GO in the UHMWPE-GO prosthesis. From these values, one can anticipate the predominance of vacancy-like defects in pristine powder GO and the predominance of sp^3^-hybridization defects in GO of the UHMWPE-GO prosthesis.^51^ While the reduction of GO was inevitable due to the high-temperature thermopressing process, the discernible increase in the *A*_D_/*A*_D’_ for the UHMWPE-GO prosthesis is indicative of very strong interactions between PE macromolecules and GO sheets.

### Thermal and Mechanical Stability Characterization

TGA curves of the UHMWPE powder, UHMWPE prosthesis, and UHMWPE-GO prosthesis show slight differences in the onset temperature of thermal decomposition, i.e., 420, 423, and 428 °C, respectively (Figure 4a). The incorporation of GO enhanced the thermal stability of the prosthesis and this could be through the strong interactions, evidenced by Raman spectroscopy. TGA curves of GO revealed four discernible thermal mass loss stages. The first stage, extending from 30 °C to 195 °C and involving ∼12% mass loss, was related to dehydration. The second stage (195–232 °C), third stage (232–525 °C), and fourth stage (525–637 °C) were attributed to the thermal decomposition of oxygen functionalities of GO with respective mass losses of ∼20%, ∼12%, and ∼38%.^32,43^ The difference between those stages was the types of functional groups undergoing thermal decomposition, as well as their degree of clustering. Indeed, the more clustered they were, the faster they decomposed and the steeper was the TGA curve.^37,52^ Notably, the remaining carbon mass of GO was ∼15% of the initial mass, whereas the remaining carbon masses of UHMWPE powder, UHMWPE prosthesis, and UHMWPE-GO prosthesis were ∼2.9%, ∼0.3%, and ∼0.0% of their respective initial hydrocarbon masses. GO oxygen-functionality mass loss was estimated to be 69.8 wt %, determined by the method outlined in ref. 53, reflecting a high degree of oxidation.

**Figure 4:**
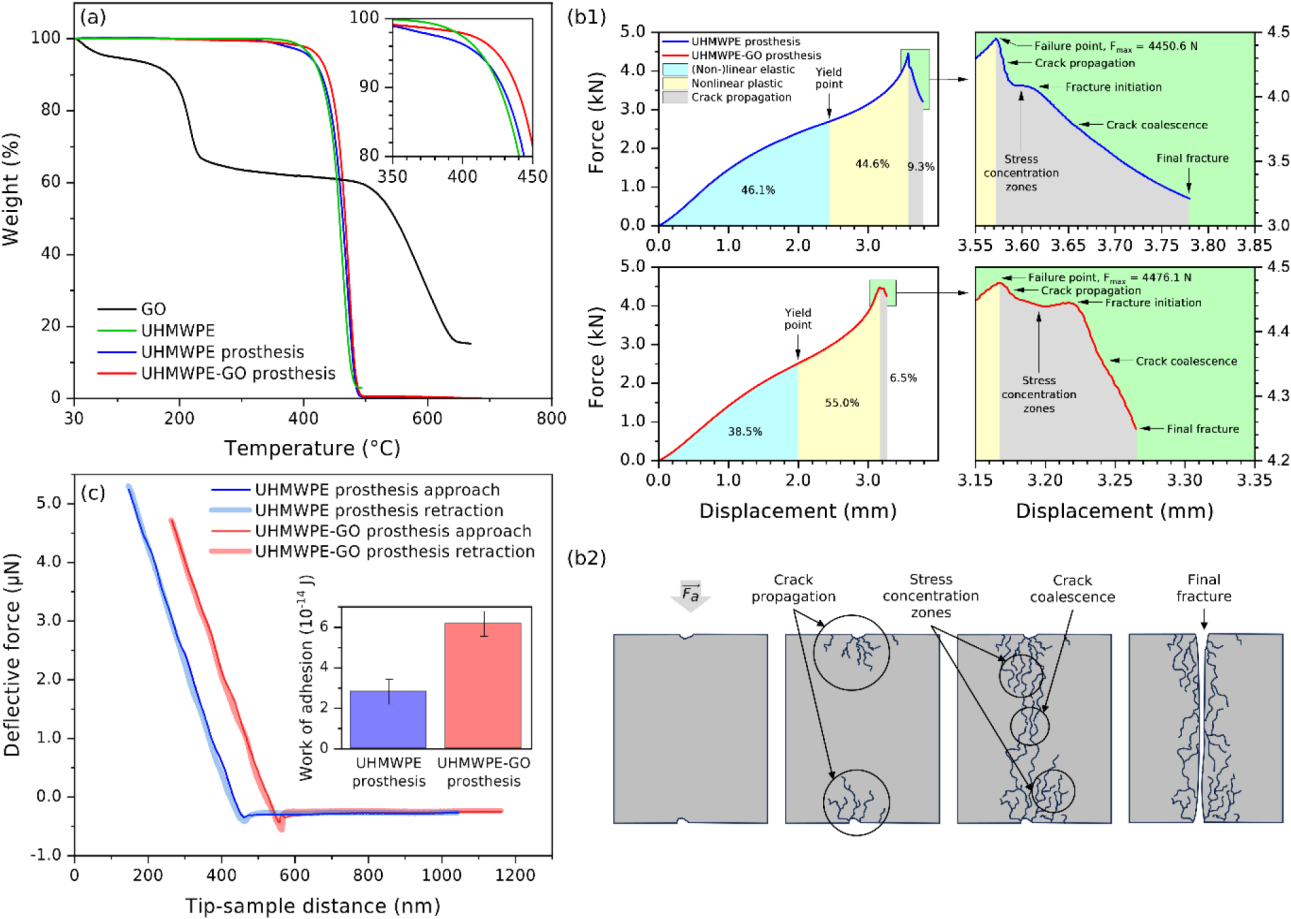
(a) TGA curves of the UHMWPE powder, GO powder, UHMWPE prosthesis, and UHMWPE-GO prosthesis with the inset showing a close-up view of the onset of thermal decomposition, (b1) force–displacement curves of UHMWPE and UHMWPE-GO prostheses with a zoomed-in view of the green-outlined apexes of the curves, (b2) schematic illustration of fracture development in prostheses to the point of final fracture (nothing to scale), and (c) AFM contact mode measurement of the UHMWPE and UHMWPE-GO prostheses with an inset showing the work of adhesion.

The force–displacement curves for the UHMWPE and UHMWPE-GO prostheses are shown in Figure 4b1. These curves consisted of elastic and plastic regions leading up to a maximum force, which marked the failure point or the onset of crack propagation.^54–57^ The maximum force for the UHMWPE-GO prosthesis was found to be higher than that of the UHMWPE prosthesis (used as the reference hereafter), indicating greater strength, as demonstrated in Figure 4b1. The area under the curve up to the failure point was the crack initiation energy, which was 8.95% lower in the UHMWPE-GO prosthesis at 7165 J/g. Specific energy was calculated by dividing the estimated area by the initial mass of the prosthesis. The area from the failure point to the final fracture point, representing the crack propagation energy, was 37.98% lower in the UHMWPE-GO prosthesis at 497 J/g. The compressive stiffness of the UHMWPE-GO prosthesis is 12.96% higher, while the yield point is 6.99% lower, estimated based on changes in curvature. Interestingly, the elastic region shrank in the UHMWPE-GO prosthesis, while the plastic deformation region expanded. A detailed analysis of the specimens beyond the failure point revealed notable variations (Figure 4b2). Crack propagation, a stress dissipation process in the UHMWPE-GO prosthesis, was brief and immediately followed by the considerable emergence of stress concentration zones.^56^ Fracture initiation occurred with characteristic crack coalescence, which was comparatively rapid in the UHMWPE-GO prosthesis, leading to the final fracture.^56^ This crack propagation model relies solely on informed interpretation of the experimental data provided by the force–displacement curves; thus, it constitutes a preliminary approach to evaluate the fracture of the prosthesis. Thorough finite element analysis of models of UHMWPE and UHMWPE-GO prostheses under loading scenarios is essential to improve the understanding of crack propagation. The force at maximum displacement was 32.29% higher in the UHMWPE-GO prosthesis at 4248.9 N. Possibly, microscale dents in the prostheses, observed by Raman microscopy and SEM, act as crack propagation centers. Images of the experimental setup for force–displacement measurement are shown in Figure S13. To further understand the adhesive nature of the surfaces of prostheses, AFM was used to probe the work of adhesion through analysis of the approach-retraction curves, as depicted in Figure 4c. The work of adhesion for the UHMWPE prostheses was estimated to be 2.82 × 10^–15^ J, while the UHMWPE-GO prosthesis exhibited more than a 2-fold increase at 6.17 × 10^–15^ J. The increased work of adhesion in the UHMWPE-GO prosthesis might enhance the bone/prostheses interface adhesion. We hypothesize that this improved adhesion could lead to better integration and stability of the prostheses to biological tissues, such as bones, connective tissue, and cartilages. Further work is essential to study the wear properties of UHMWPE and UHMWPE-GO prostheses, characterize wear debris, and investigate wear debris potential to elicit an immune response.

### Cell/Surface Interaction

Finally, a study of the interaction of the new prosthesis with mammalian cells was performed following physical-chemical and mechanical characterization. For this purpose, human fibroblasts (L132 cell line) were cultured on the prosthesis surface. To assess the biological effects of each compound, we first performed solution-based viability assays to isolate the individual biocompatibility effects of GO and UHMWPE before composite fabrication.

In terms of results, as shown in Figure 5, the cells exhibited distinct morphological characteristics depending on the surface on which they were cultured. The fibroblast L132 cell line demonstrated more robust growth on the graphene-based prosthesis UHMWPE-GO (Figure 5a–b), compared to reduced growth on the UHMWPE prosthesis. In fact, earlier studies showed that reduced graphene oxide can improve biocompatibility.^24,58^ These findings also suggested enhanced cell adhesion and good biocompatibility with the UHMWPE-GO (Figure S14), as previously documented with mesenchymal cells and silicon substrate.^59,60^ Furthermore, the cytoskeleton morphology of cells on the graphene-modified prosthesis exhibited a more organized structure, indicating that this surface enhances cell interaction, adhesion, and could be beneficial for implants.^10,61^

**Figure 5:**
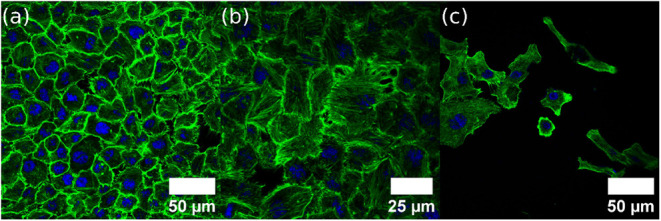
(a) Confocal images showing L132 cells on the UHMWPE-GO prosthesis with a close-up view (b) and L132 cells on the UHMWPE prosthesis (c).

For this point, cell adhesion improvement is a promising result since it indicates that the UHMWPE-GO appears to facilitate the adhesion of these cells derived from human fibroblasts, as previously shown in UHMWPE reinforced with carbon.^10^ Regarding cell biology, the cytoskeleton plays a crucial role in cell adhesion by providing structural support and facilitating the transmission of mechanical signals between the cell and its extracellular environment.^62^ Organized cytoskeletal structures, such as actin filaments, contribute to the formation and stabilization of focal adhesions, specialized sites where cells attach to the substrate.^63^ These focal adhesions not only anchor the cell but also regulate cell movement, shape, and signaling pathways.^64^ Proper cytoskeletal organization is, therefore, essential for robust cell adhesion, influencing cell behavior and interaction with the surrounding environment.^64,65^ Works from other groups have already shown good cell proliferation and adhesion on material containing graphene.^22,66,67^ In addition, the wear reduction rate can be achieved by increasing adhesion forces and hydrophobicity.^67^ Graphene is also known to have good conductivity, and cells can benefit.^68^ Better growth in the UHMWPE-GO prosthesis might also have been enhanced because of the conductivity characteristic of reduced GO.^69–71^ Although the UHMWPE-GO prosthesis demonstrated better proliferation and adhesion of fibroblasts, other analogues showed antiproliferative activity in other cells, as demonstrated in other studies.^10,71,72^

Electrostatic force microscopy (EFM) enabled visualization of surface charge distribution on the surfaces of UHMWPE and UHMWPE-GO prostheses (Figure 6). EFM phase images reflected the electrostatic interactions between the biased tip and the sample surface: higher phase values indicated repulsive forces, whereas lower phase values corresponded to attractive forces.^73^ In the case of the UHMWPE sample, the EFM phase image exhibited minimal contrast, suggesting a uniform distribution of surface charge, predominantly positive, as expected from the nonpolar, insulating nature of polyethylene. The homogeneity of the phase signal across the imaged area confirmed the electrostatic uniformity of the UHMWPE surface. Conversely, the EFM phase image of UHMWPE-GO revealed clear phase contrast, indicative of a nonuniform surface charge distribution. Regions of elevated phase signal coexisted with areas of reduced signal, reflecting a spatial variation in the electrostatic forces experienced by the probe. This heterogeneity was attributed to superficial GO agglomerates, which protruded from the polymer matrix and exposed negatively charged functional groups of graphene oxide to the surface. These negatively charged regions contrast sharply with the positively charged UHMWPE matrix, forming localized surface potential gradients. The presence of such electrostatic gradients can be significant from a biomedical perspective. Previous studies have demonstrated that surface potential differences can stimulate cell proliferation by mimicking endogenous bioelectric signals.^74^ In line with this, L132 fibroblast cells cultured on UHMWPE-GO prostheses exhibited enhanced adhesion and proliferation compared to cells on GO-free UHMWPE prostheses, as shown in Figure 5. This observation suggests that the endogenous electric field (EEF) generated by the UHMWPE-GO composite may replicate the natural bioelectric field of bone tissue, thereby promoting osteointegration.^75^

**Figure 6:**
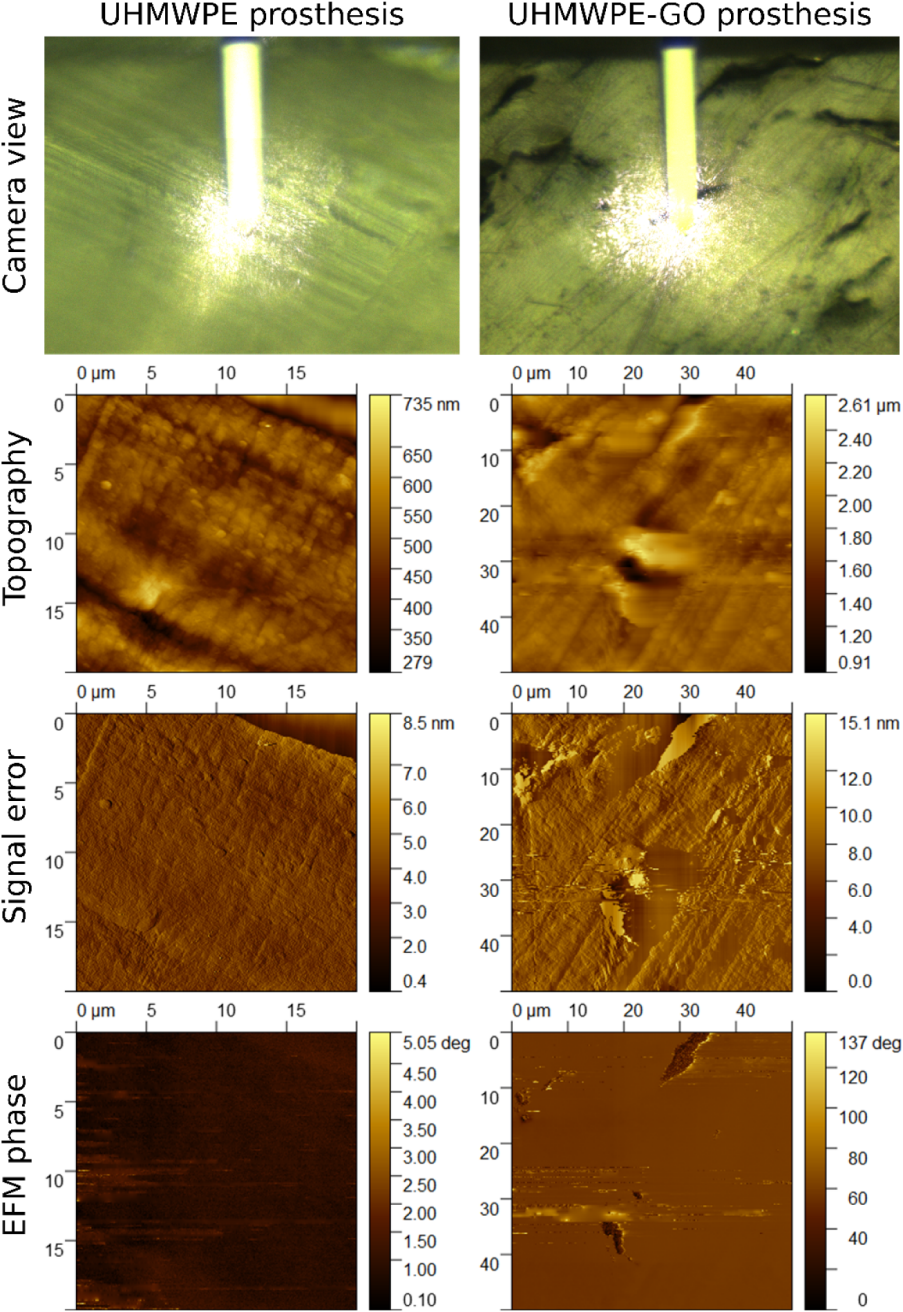
EFM characterization of prostheses made of UHMWPE and UHMWPE-GO. EFM phase images present contrasting regions in the case of the UHMWPE-GO prosthesis, which confirms the presence of surface potential gradients.

*Candida species* are the main contributor to fungal nosocomial infections that can evolve into sepsis, particularly in intensive care units.^76^ The ability to form biofilms on catheters and prostheses presents a major threat to public health.^77^ Although *C. albicans* is the most frequent sepsis-causing agent, nonalbicans species represent a growing threat.^78,79^ In the context of the growing selection of isolates that are tolerant or resistant to antifungal drugs, and the lack of new therapeutic agents, the research on alternative materials or compositions that minimize or prevent the proliferation of biofilm-forming microbes is highly envisaged for the manufacturing of prostheses and other medical devices.^80,81^

Our data indicate that the combination of GO and UHMWPE efficiently impaired *C. tropicalis* cell viability (Figure S15), proliferation (Figure S16) and surface colonization (Figures S17–S19). Regarding viability tests, a more pronounced inhibition was achieved when the mixture of GO and UHMWPE at 48 mg/mL (in medium) was present in the culture broth. For *C. albicans* yeasts, the detected inhibitory effects were only due to the presence of ethanol in GO and UHMWPE powders dispersed in alcohol (0.6 mg/mL). *C. glabrata*viabilities were mostly impacted by GO and UHMWPE in alcohol, but alcohol alone had no significant effect (Figure S15). The concentrations with powders dispersed in alcohol were the same as those used in the murine cell viability test (Figure S14).

Since *C. tropicalis* was the only species whose cell viability was affected by the mixture of GO and UHMWPE, we proceeded with the following tests only with *C. tropicalis*. On the one hand, the 12 mg/mL GO concentration did not impact *C. tropicalis* number of colonies. On the other hand, increasing concentrations of UHMWPE provoked a significant reduction in the number of colonies (Figure S16). In addition, UHMWPE-GO had a higher contact angle, consequently increasing the hydrophobicity, which in turn may have increased the antifungal effect.^82^ Furthermore, the wrinkle morphology and negative load of GO can influence the reduction of adhesion of *Candida* cells to the GO-prosthesis.^83–85^ These results are impactful due to the increasing data on the morbidity and mortality rates associated with this fungus, as recently reviewed by Keighley et al. (2024),^79^ including medical devices.^79,81^

Table S3 provides a comparison between different approaches for the fabrication of UHMWPE composites with key characteristic descriptors. It is worth mentioning that while several descriptors are lacking for selected studies rendering the comparison partly inconclusive, the pairwise comparison between pure matrix UHMWPE and composite UHMWPE can inform about the hybrid effect of the fabrication process. This table provides future studies with valuable descriptors that will help fathom the hybrid effect concomitant with carbon nanomaterial fillers. One key descriptor is the percent crystallinity (X_C_). Our fabrication method results in a 7.90% increase in crystallinity of the UHMWPE-GO composite compared to the UHMWPE pure matrix. The fabrication method of F. Mindivan and A. Çolak^86^ results in 4.54% increase in crystallinity of the UHMWPE/RGOC-1 biocomposite compared to the UHMWPE pure matrix, a relative increase that is smaller despite utilizing ethanol. The UHMWPE/Gr 60 nm composite multistep dry fabrication method of Cheng-Ying Liu et al.^87^ results in ∼11% increase in crystallinity compared to the UHMWPE pure matrix, a relative increase that is discernibly larger, but necessitates octa-screw extrusion of precursors into strips, grinding strips into powders, and ultimate molding. Therefore, our solvent-free one-step method of fabrication is facile and yields the UHMWPE composite with improved crystallinity, thermal and mechanical stability, potential for EEF generation, and enhanced interactions with mammalian cells.

Future studies should verify the stability of this UHMWPE-GO prosthesis production process and the effect over time on an implanted person, with respect to regulatory aspects such as ISO 10993.

## Conclusion

Based on our extensive research and experimental analysis, we successfully developed a novel protocol for incorporating 1.0 wt % into UHMWPE in one solvent-free step, directly into the mold, to engineer a new medical prosthetic material. Through comprehensive characterization, we have demonstrated that the UHMWPE-GO composite exhibits significant improvements in both physicochemical and biological properties, compared to conventional UHMWPE. The integration of GO not only enhances the mechanical and thermal stability of the prostheses but also improves its surface properties, including reduced roughness, increased hydrophobicity, and EEF generation. This suggests a successful integration of the graphene’s hydrophobic nature into the composite material. Importantly, the UHMWPE-GO prostheses demonstrated superior interactions with mammalian cells, as human blasts exhibited enhanced adhesion and cytoskeleton organization on the graphene-modified surfaces. These findings highlight the potential of prostheses for better cell adhesion and biocompatibility, crucial traits for effective integration into biological systems, such as bone, connective tissue, and cartilage. Together, these results underscore the promise of UHMWPE-GO prostheses in medical applications, offering improved performance and biocompatibility.

## Supplementary Material



## Notes

Characterization of powders of GO and UHMWPE (SEM, XRD); results of SEM/EDS, AFM, contact angle measurements, Raman spectroscopy, force–displacement measurements of UHMWPE and UHMWPE-GO prostheses; biocompatibility of powders of GO and UHMWPE; cell viability assessment in the presence of powders of GO and UHMWPE; PFU test with powders of GO and UHMWPE; powders and prostheses swab (PDF)
