# Supplementary material for: Graphene Oxide Composites with Ultrahigh-Molecular-Weight Polyethylene for Innovative Prostheses for Arthroplasty
Source: ACS Appl Bio Mater. 2025 Dec 15;9(1):81–95. doi: 10.1021/acsabm.5c01289 (PMC12776581; doi:10.1021/acsabm.5c01289)
Supplement: Supplementary file 1 [file mt5c01289_si_001.pdf]

Supporting Information

Graphene Oxide Composites with Ultra-High-Molecular-Weight Polyethylene for Innovative Prostheses for Arthroplasty

*Ahmed Subrati[1‡], Thyago Arruda Pacheco[2‡], Ítalo Azevedo Costa[3], Victor Carlos Mello[2], John Fredy Ricardo Marroquin[4], Mikołaj Kościński[5,6], Ludmila Alvim Gomes Pinho[7], Ariane Pandolfo Silveira[2], Jorlandio Felix[4], Marcilio Cunha-Filho[7], Marcio José Poças-Fonseca[2], Ricardo Bentes de Azevedo[2], Sonia Nair Báo[2], Rander Pereira Avelar[8], Leonardo Giordano Paterno[3], Sergio Moya[1], João Paulo Figueiró Longo[2]**

1- Centro de Investigación Cooperativa en Biomateriales (CIC biomaGUNE), 20009 Donostia-San Sebastián, Guipúzcoa, Spain

2- Institute of Biological Sciences, University of Brasilia, Brasilia 70910-900, Brazil

3- Polymer and Nanomaterials Research Laboratory, Chemistry Institute, University of Brasilia, Brasilia, DF 70910-900, Brazil

4- LabINS BSS 297, Institute of Physics, University of Brasília, Brasília, 70910-900, Brazil

5- Faculty of Physics and Astronomy, Adam Mickiewicz University, Uniwersytetu Poznańskiego 2, Poznań, 61-614, Poland

6- Department of Physics and Biophysics, Faculty of Food Science and Nutrition, Poznań University of Life Sciences, Wojska Polskiego 38/42, Poznań, 60-637, Poland

7- Laboratory of Food, Drug and Cosmetics (LTMAC), School of Health Sciences, University of Brasília, Brasília, DF, 70910-900, Brazil

8- CPMH Digital, Brasília, DF, 71200-260, Brazil

[‡]These authors contributed equally.

*Corresponding author: João Paulo Figueiró Longo, E-mail address: jplongo82@gmail.com.

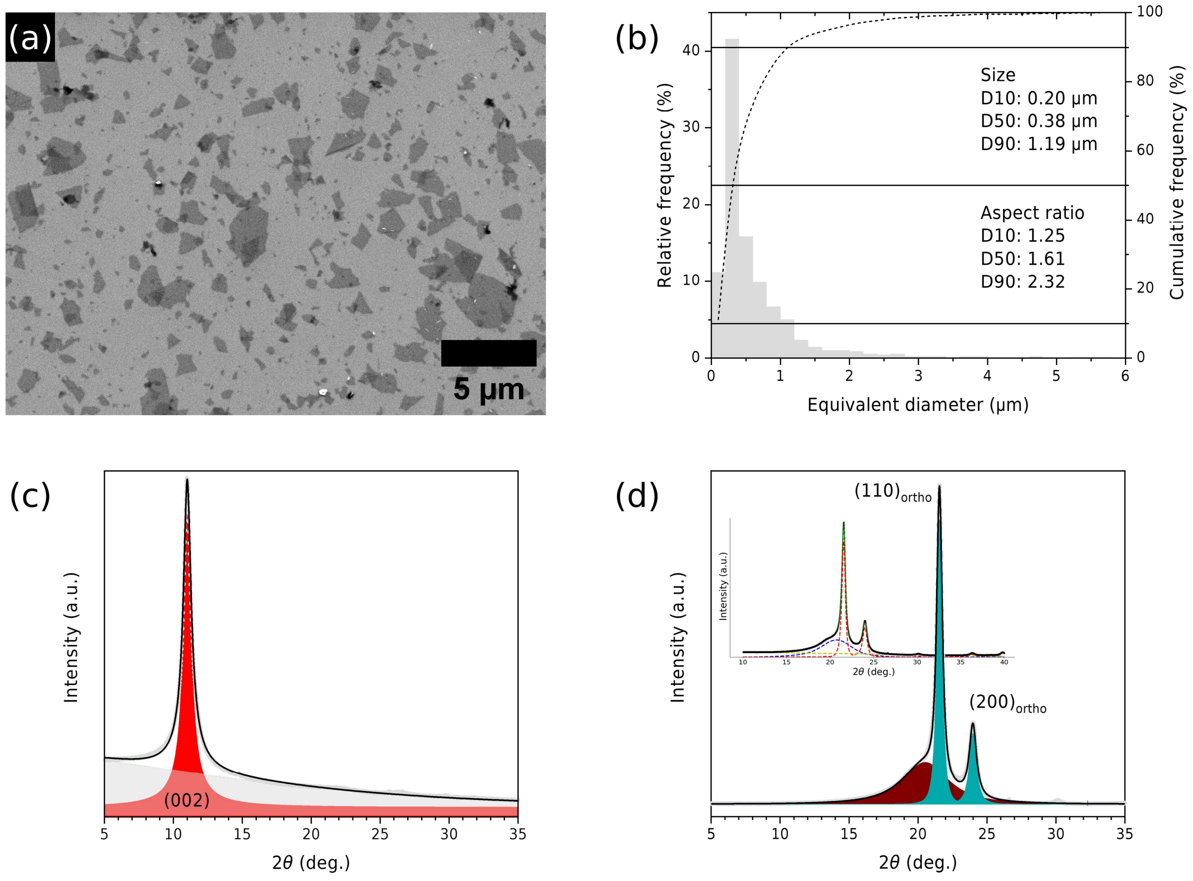

Figure S1. Characterization of powders GO and UHMWPE: (a) SEM image of GO sheets on a Si wafer chip, (b) size distribution of GO sheets with summarized statistical analysis of size and aspect ratio of sheets, (c) XRD pattern of GO powder, and (d) XRD pattern of UHMWPE powder and associated AMORPH analysis and deconvolution.

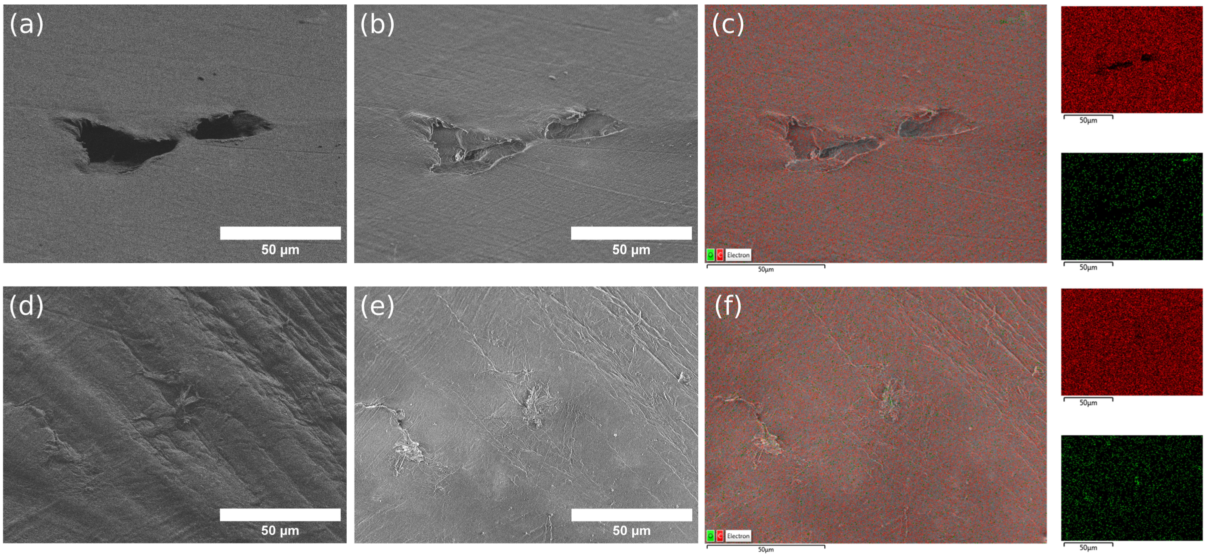

Figure S2. UHMWPE prosthesis SEM/EDS analysis: (a) secondary electron detector (SED) image, (b) backscattered electron detector image (BED), and (c) EDS C/O elemental layered image with corresponding C (red) and O (green) elemental maps shown as insets. UHMWPE-GO prosthesis SEM/EDS analysis: (d) SED image, (e) BED image, and (f) EDS C/O elemental layered image with corresponding C (red) and O (green) elemental maps shown as insets.

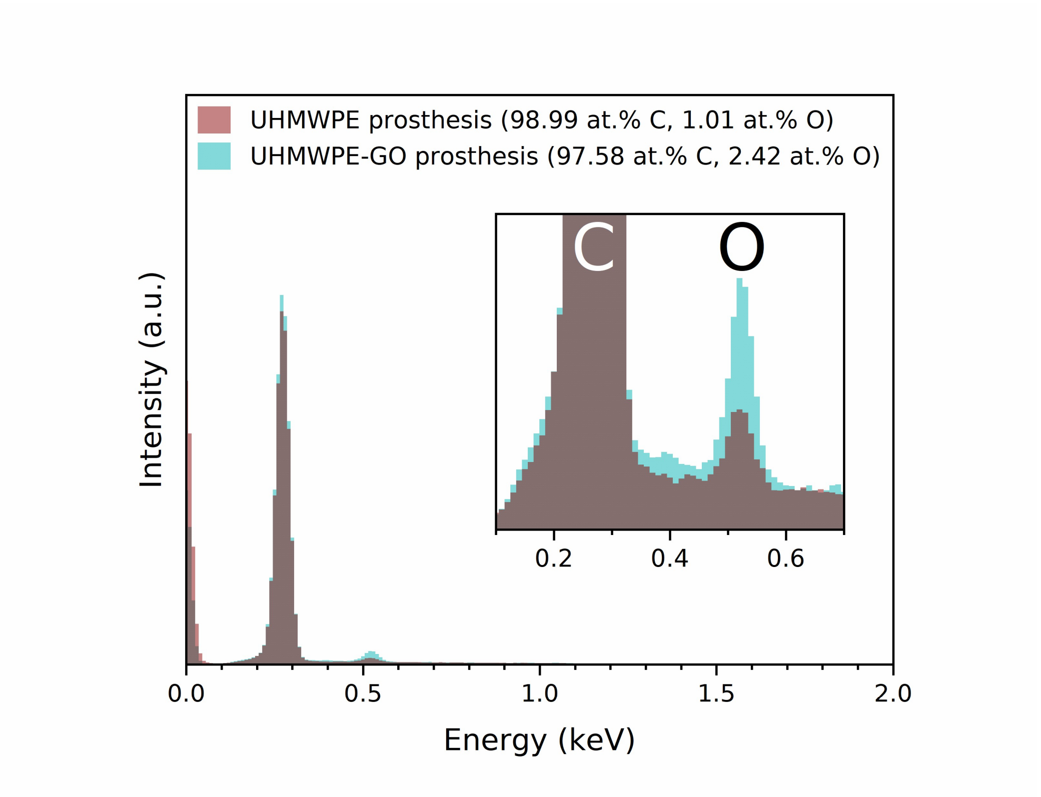

Figure S3. Energy dispersive X-ray spectra of UHMWPE prosthesis (teal) and UHMWPE-GO prosthesis (wine).

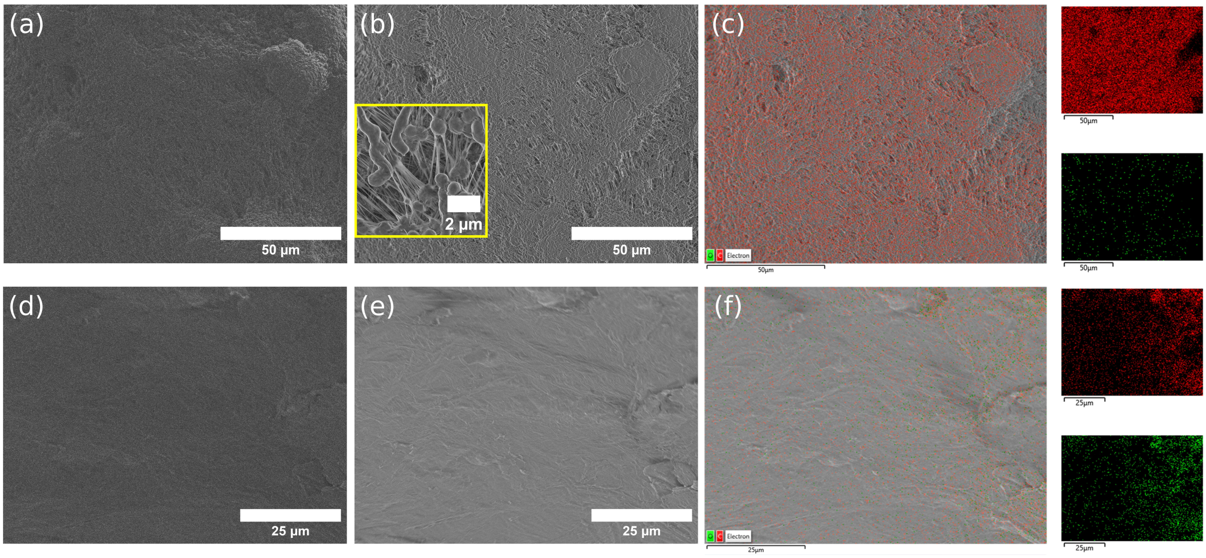

Figure S4. UHMWPE powder SEM/EDS analysis: (a) SED image, (b) BED image with yellow-outlined inset showing the network of fibrillar bundles and lamellar noduli, and (c) EDS C/O elemental layered image with corresponding C (red) and O (green) elemental maps shown as insets. GO powder SEM/EDS analysis: (d) SED image, (e) BED image, and (f) EDS C/O elemental layered image with corresponding C (red) and O (green) elemental maps shown as insets.

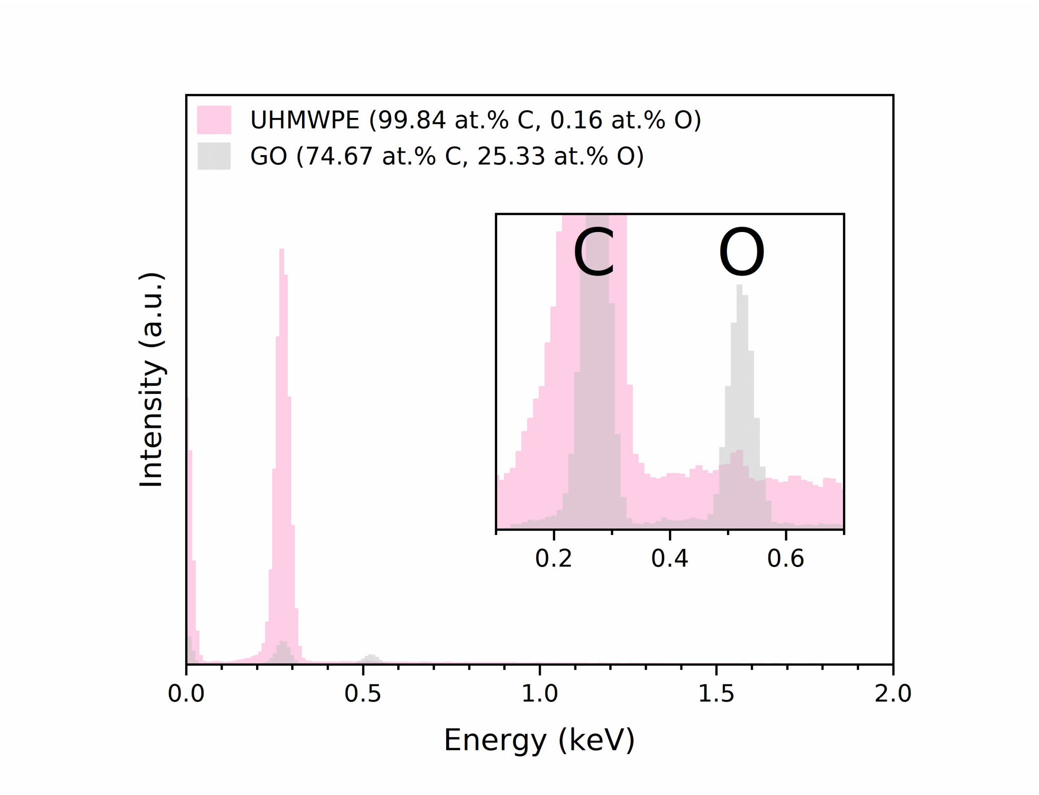

Figure S5. Energy dispersive X-ray spectra of UHMWPE powder (light pink) and GO powder (light grey).

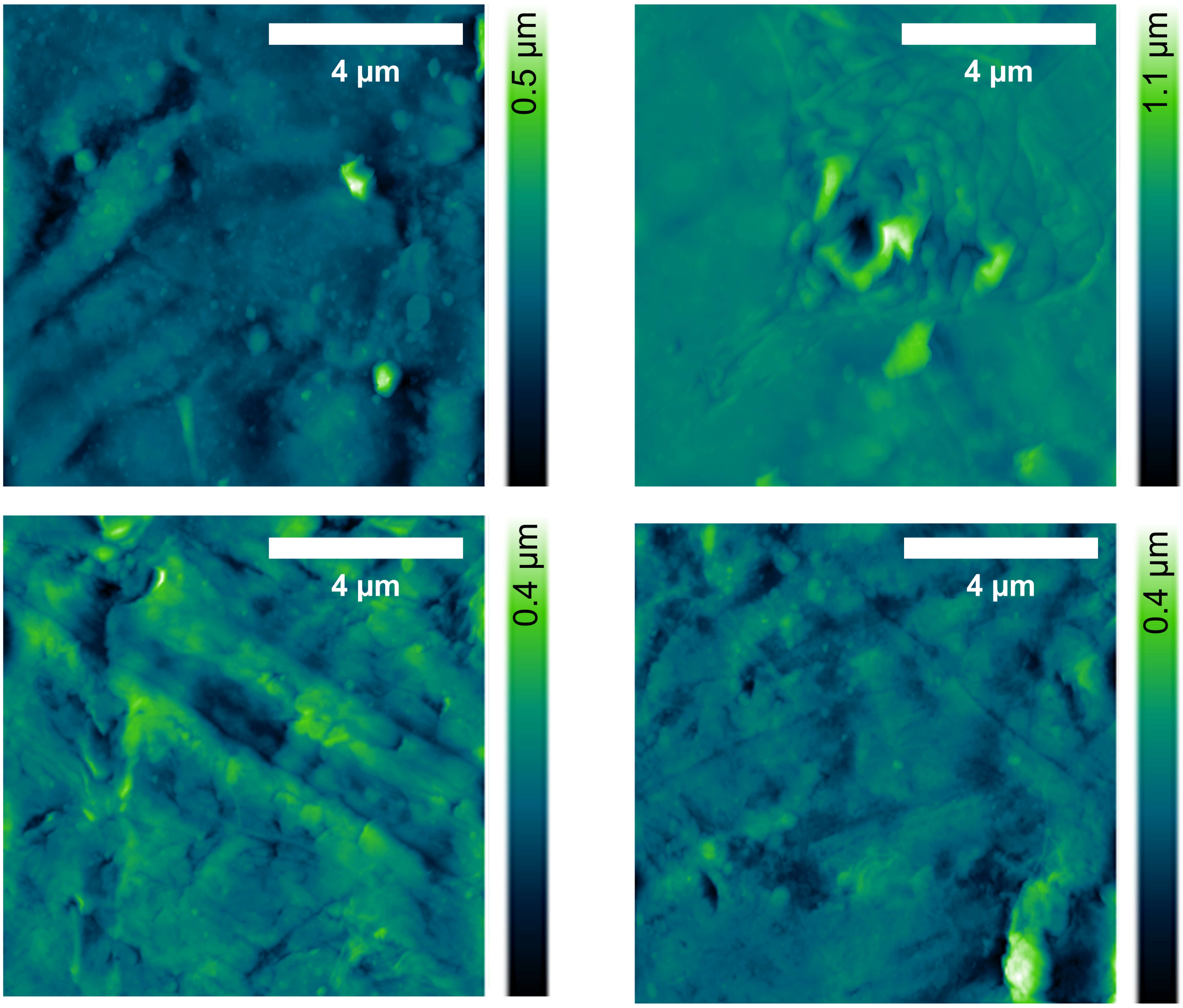

Figure S6. AFM images of the UHMWPE prosthesis (top) and the UHMWPE-GO prosthesis (bottom) showing smooth (left) and irregular (right) topographical sections.

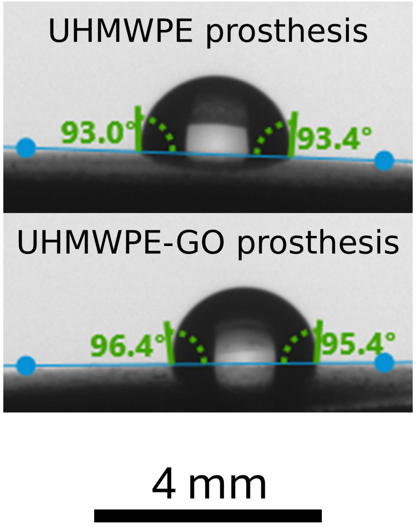

Figure S7. The contact angle measurements of the UHMWPE and UHMWPE-GO prostheses.

Table S1. Summary of the deconvolution of C 1s X-ray photoelectron spectra of UHMWPE prosthesis, UHMWPE powder, GO powder, and UHMWPE-GO prosthesis. B.E.: binding energy and at.%: atomic percentage.

**Components**	**UHMWPE prosthesis**		**UHMWPE**		**GO**		**UHMWPE-GO prosthesis**	
	B.E. (eV)	Content (at.%)	B.E. (eV)	Content (at.%)	B.E. (eV)	Content (at.%)	B.E. (eV)	Content (at.%)
C=C	-	-	-	-	285.0	27.1	284.0	20.8
C–C	285.5	100.0	285.7	71.6	285.8	11.2	286.0	54.2
C–O	-	-	287.3	28.4	287.0	37.9	287.2	22.1
C=O	-	-	-	-	288.3	14.4	288.3	1.3
COO	-	-	-	-	289.4	7.2	289.4	1.6
π–π	-	-	-	-	291.0	2.2	-	-
Total C (at.%)	98.5		97.4		71.0		95.7	
Total O (at.%)	1.5		2.6		29.0		4.3	
C/O (at.%/at.%)	64.6		37.2		2.4		22.0	

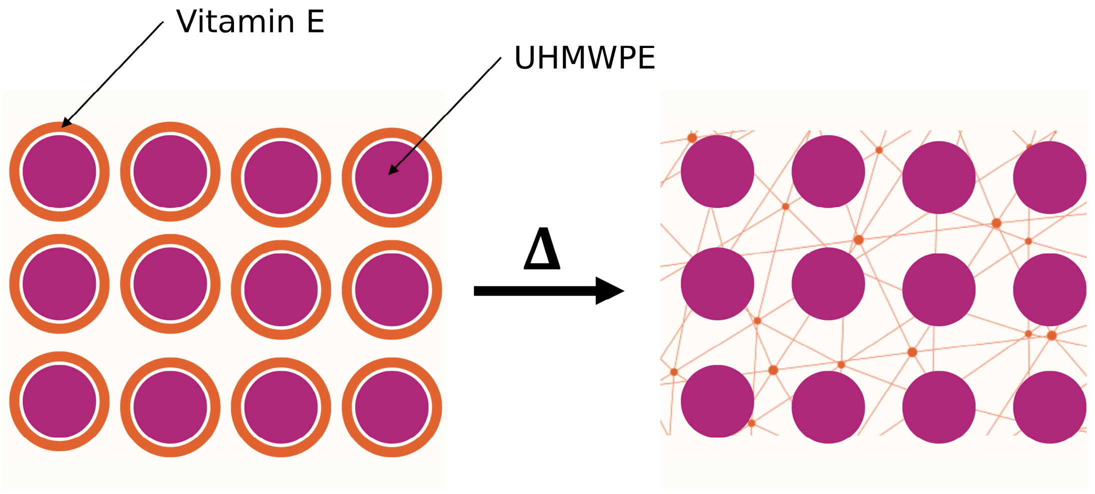

Figure S8. The plasticizing effect of vitamin E in the rendering process of the UHMWPE and UHMWPE-GO prostheses.

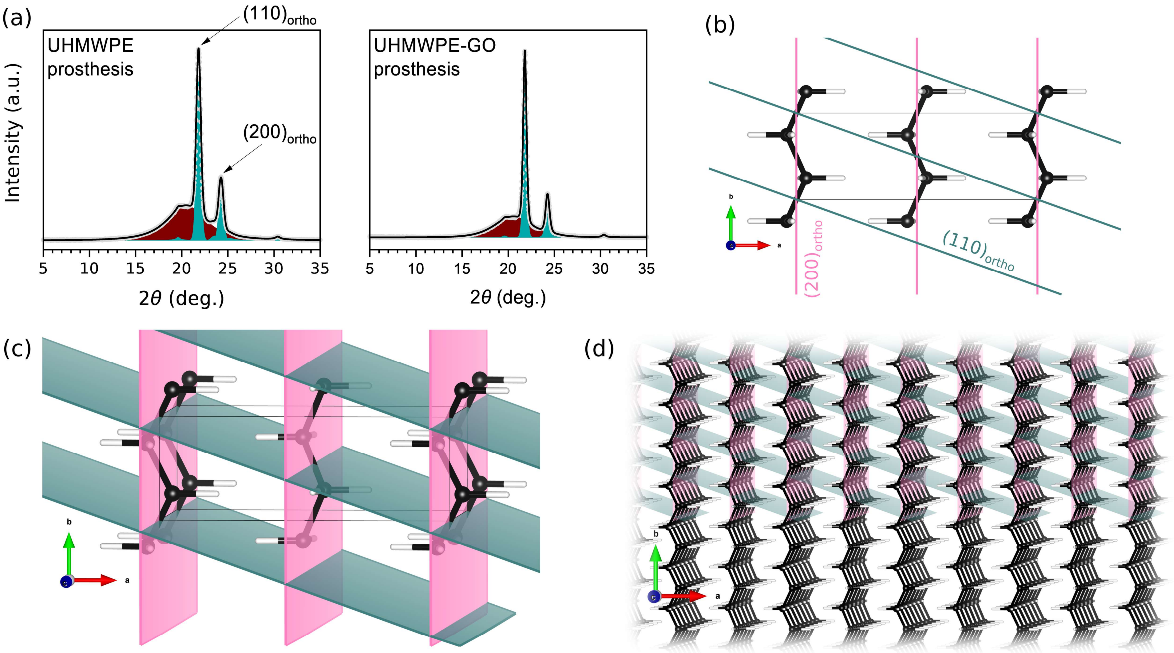

Figure S9. (a) Deconvolution of the XRD patterns of the UHMWPE and UHMWPE-GO prostheses. The crystalline and amorphous domains are represented in colors teal and wine, respectively. The orthorhombic peaks (110) and (200) were used to estimate the transverse crystallite size (D_{110}) and longitudinal crystallite size (D_{200}) from 2θ position and width information. (b) Unit cell of polyethylene (data retrieved from the Materials Project for H_2C, mp-985782, from database version v2023.11.1) depicting the orthorhombic planes (110) and (200) in teal and pink, respectively. (c) The unit cell viewed in (b) but in another projection allowing a better view of the planes. (d) An expanded structure to illuminate the periodicity in the orthorhombic crystalline phases in the UHMWPE and UHMWPE-GO prostheses.

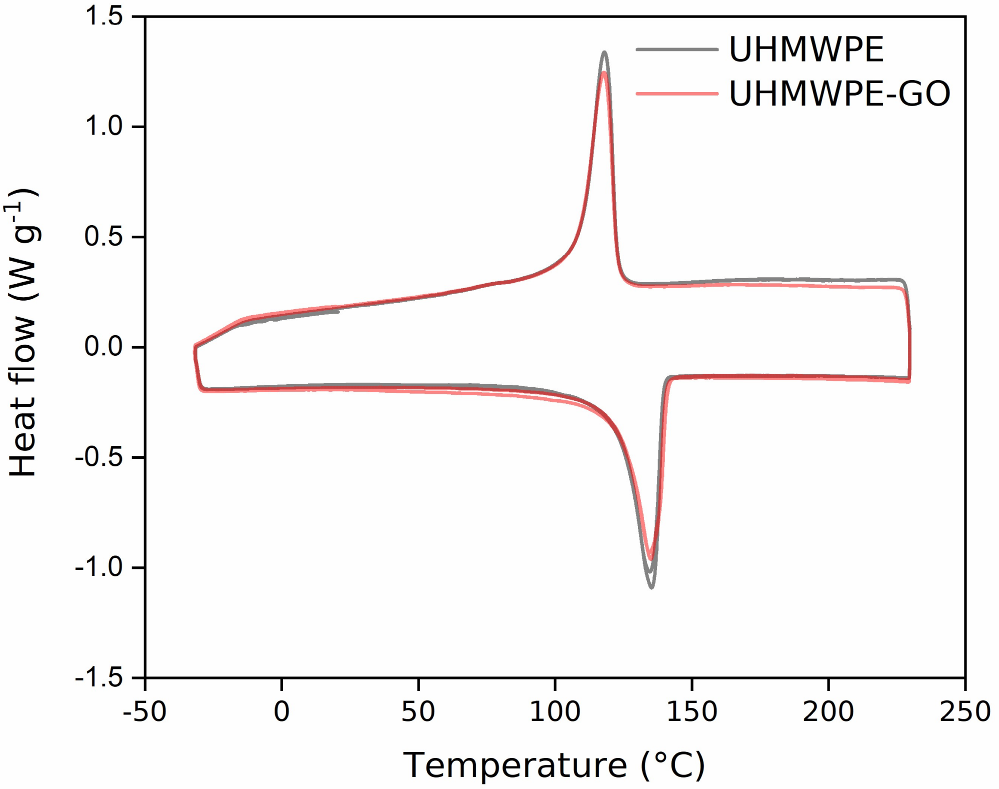

Figure S10. DSC curves of UHMWPE and UHMWPE-GO prostheses. Exothermic down.

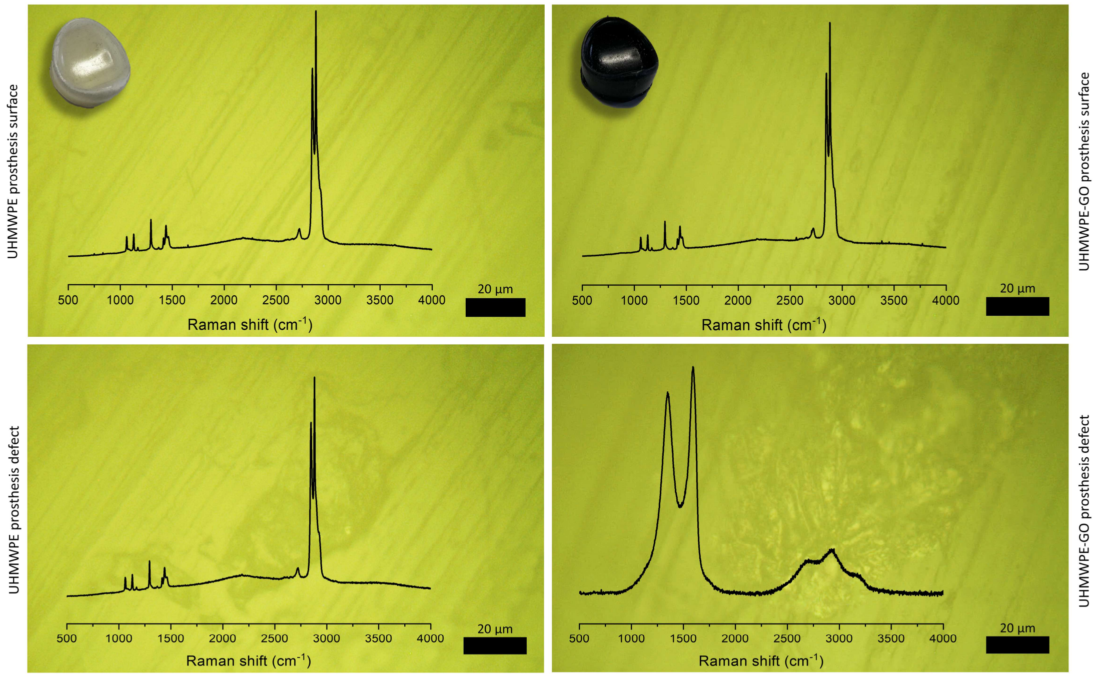

Figure S11. Raman spectra of (top) surfaces of UHMWPE and UHMWPE-GO prostheses and (bottom) defects depicting the strong dependence of GO characteristic bands emergence on surface topography.

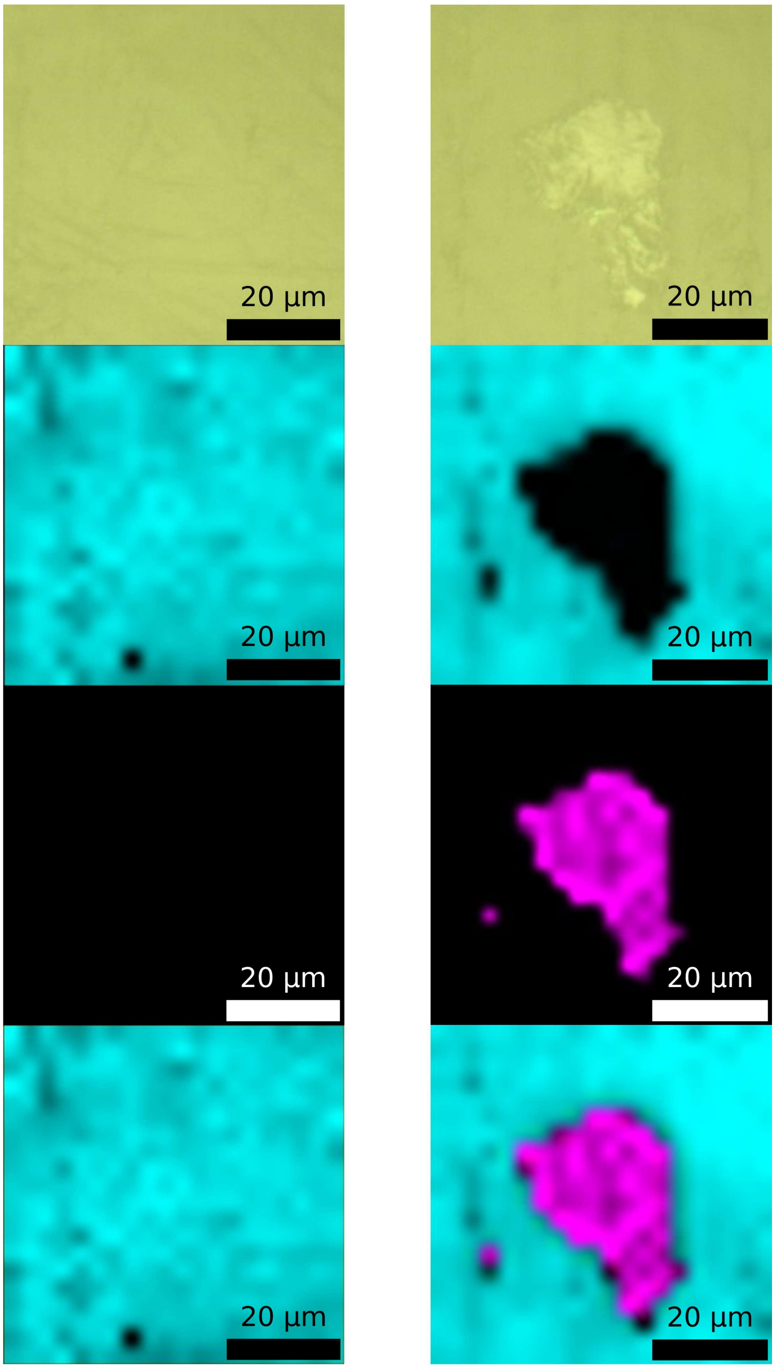

Figure S12. Raman maps of the smooth (left) and defective (right) surfaces of the UHMWPE-GO prosthesis. From top to bottom: surface image, UHMWPE signal map (2790-2980 cm^{-1}) in cyan, GO signal map (900-1900 cm^{-1}) in magenta, and overlay image.

Table S2. Summary of the deconvolution of Raman spectra obtained for GO powder and GO in the UHMWPE-GO prosthesis. FWHM: full-width-half-maximum.

**Sample**		**Band**					
		**D***	**D**	**D"**	**G**	**D'**	**M**
**GO**	Intensity	99	2577	569	2001	1307	103
	FWHM (cm^{-1})	61	78	134	31	19	34
	Area (cm^{-1})	18924	631932	95215	195658	78800	10975
	Center (cm^{-1})	1119	1354	1529	1586	1612	1771
**GO in UHMWPE-GO prosthesis**	Intensity	96	2500	600	2037	1021	41
	FWHM (cm^{-1})	76	70	129	28	15	18
	Area (cm^{-1})	22714	553023	96665	177316	48769	2318
	Center (cm^{-1})	1164	1350	1534	1588	1616	1754

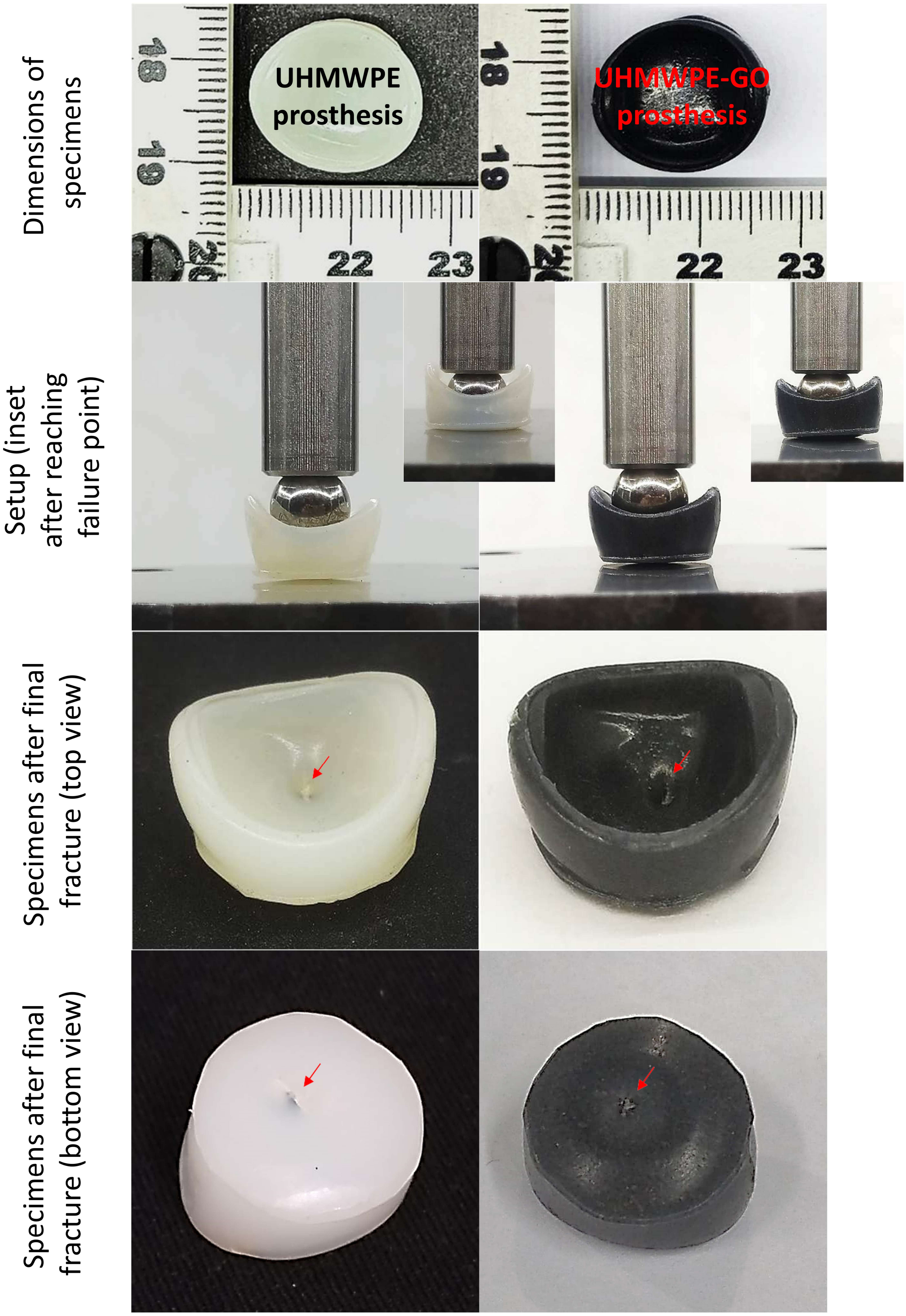

Figure S13. Photographs of the UHMWPE and UHMWPE-GO prostheses and experimental setup for the force-displacement measurements.

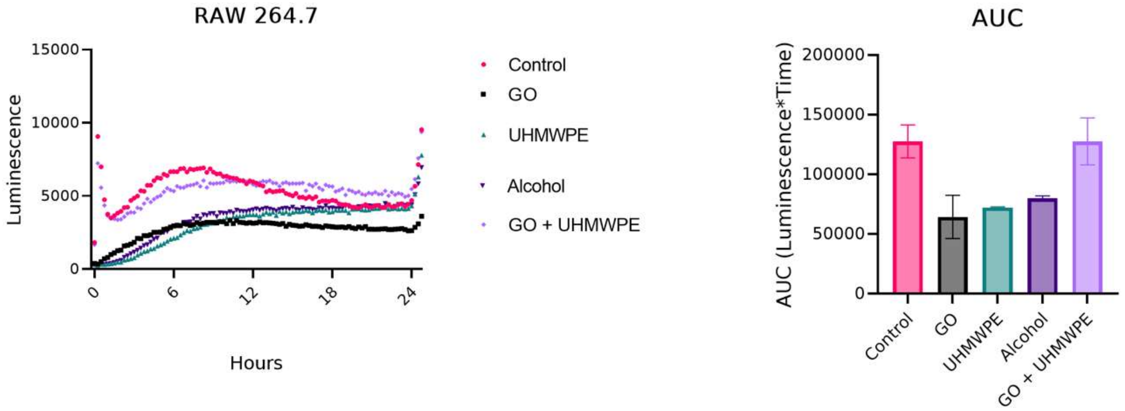

Figure S14. Biocompatibility of the UHMWPE and GO powders (0.6 mg/mL in alcohol) with RAW 264.7 and 1% GO (w/w) with UHMWPE in medium.

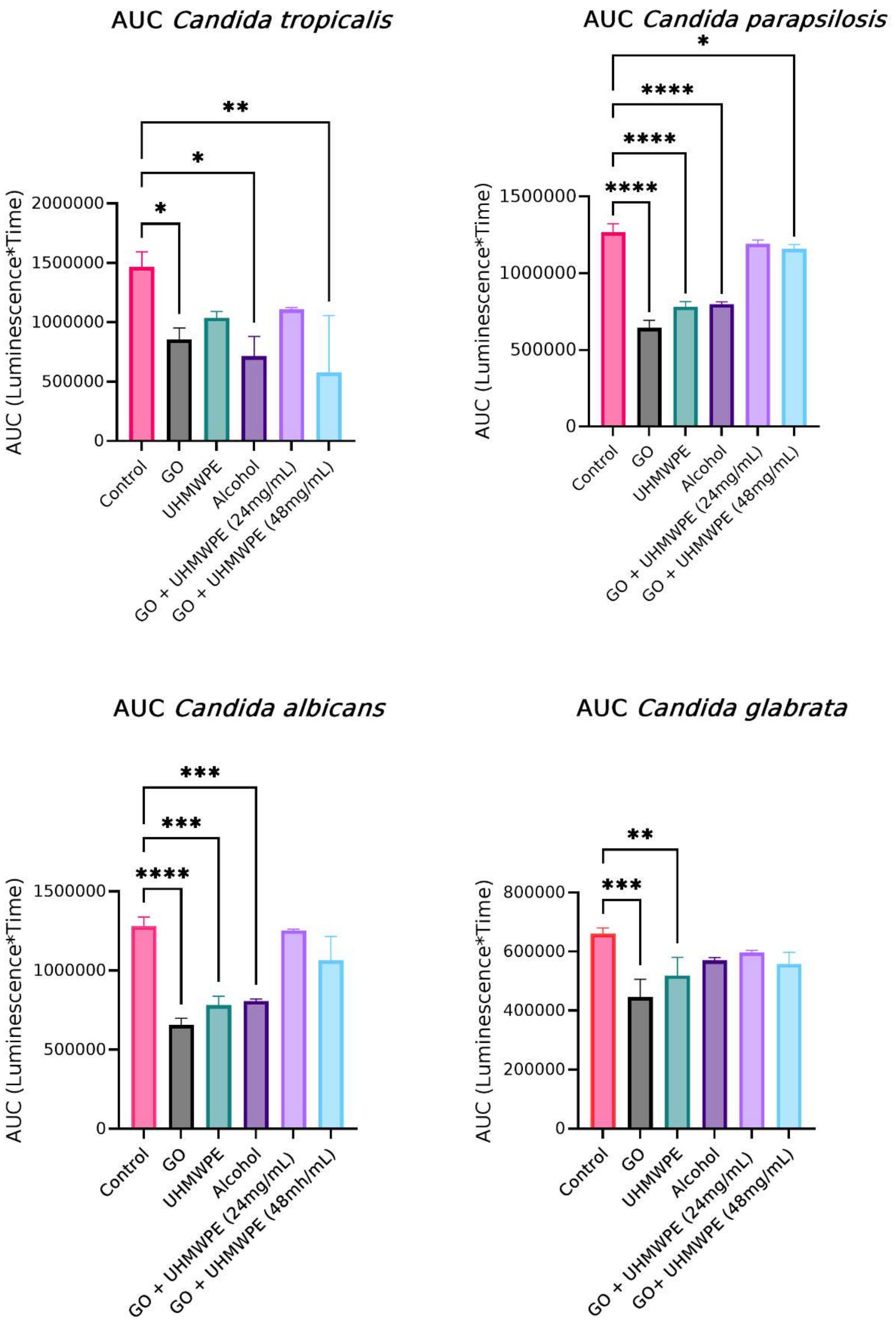

Figure S15. Cell viability assessment for *C. tropicalis, C. albicans, C. parapsilosis and C. glabrata* in the presence of GO, UHMWPE (0.6 mg/mL in alcohol) or in a mixture of them, but GO being 1% (w/w) in medium.

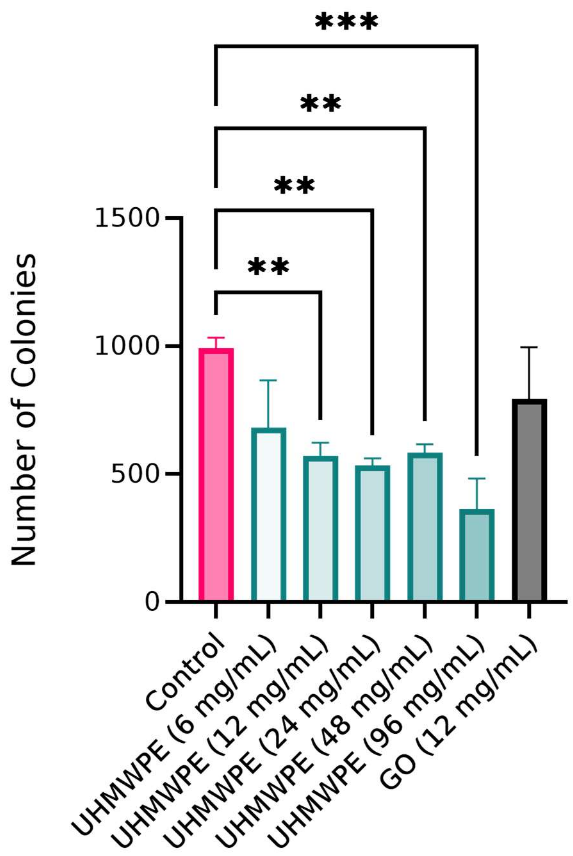

Figure S16. Colony forming units counting for C. tropicalis grown on MHA plates supplemented with different concentrations of UHMWPE or 12 mg/mL graphene in medium.

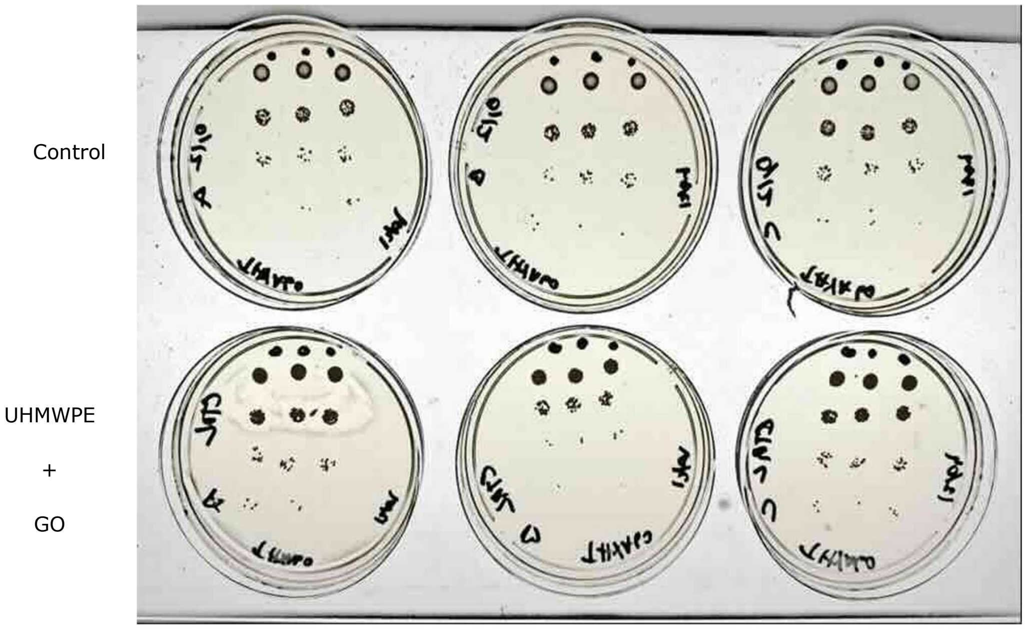

Figure S17. PFU test with UHMWPE and GO powders in *Candida tropicalis*. Non-significant.

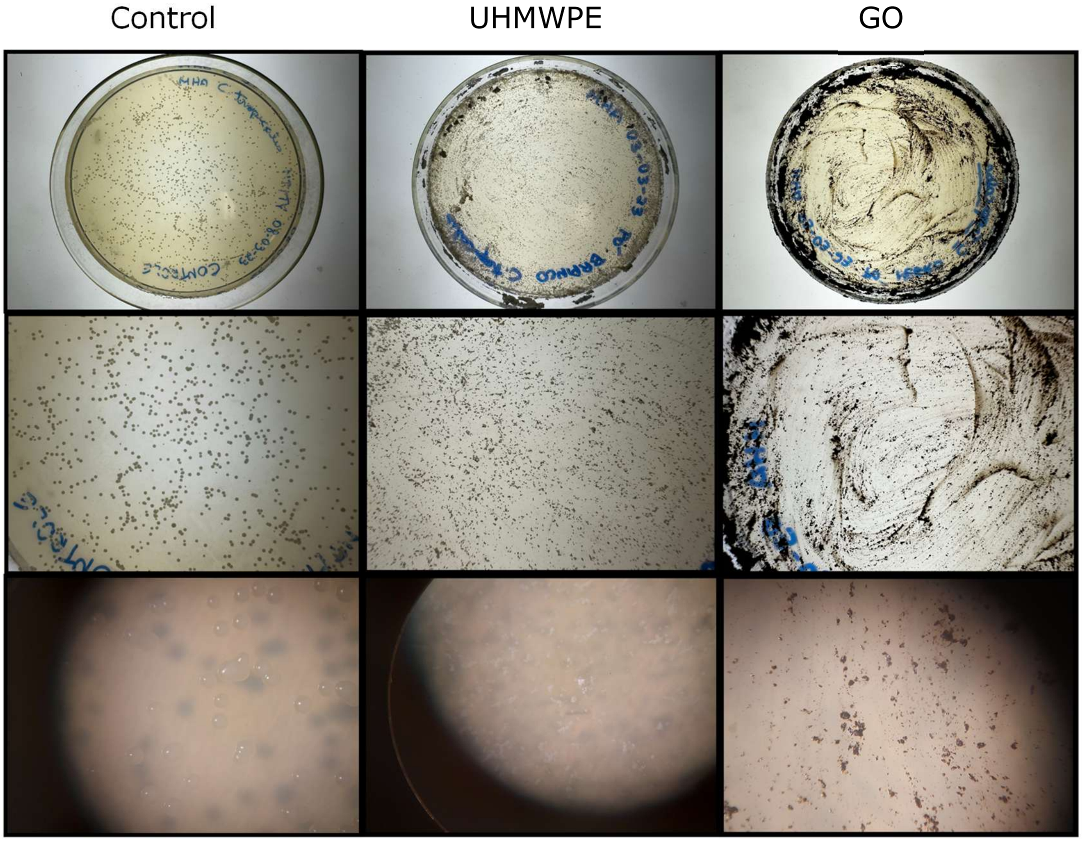

Figure S18. *Candida tropicalis* swab and UHMWPE and GO powders.

UHMWPE

UHMWPE-GO

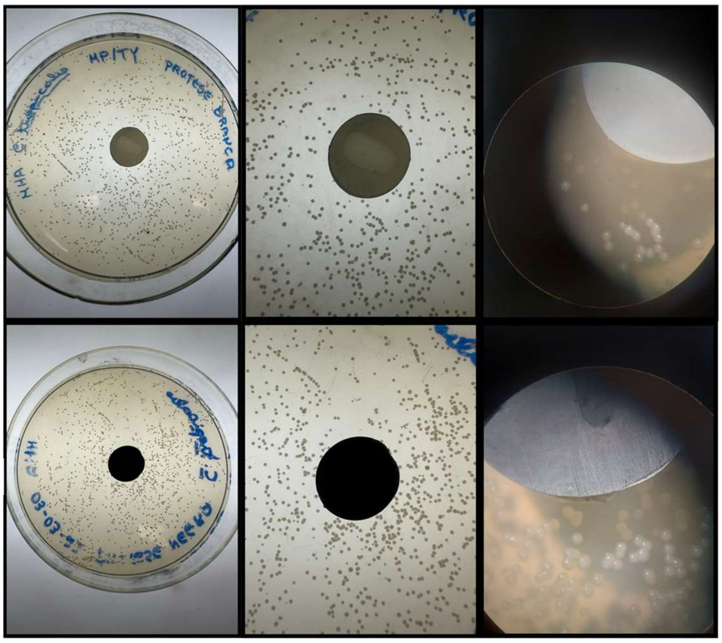

Figure S19. *Candida tropicalis* swab and UHMWPE and UHMWPE-GO prostheses.

Table S3. Comparison between different UHMWPE composite rendering methods.

Material	UHMWPE prosthesis	UHMWPE-GO prosthesis	UHMWPE pure matrix composite	UHMWPE/RGOC-1 biocomposite	UHMWPE pure matrix composite	UHMWPE/Gr 8 nm	UHMWPE/Gr 60 nm
Matrix	UHMWPE (3.5 x 10^6 g mol^{-1}); enriched with vitamin E	UHMWPE (3.5 x 10^6 g mol^{-1}); enriched with vitamin E	UHMWPE	UHMWPE	UHMWPE	UHMWPE (3.2 x 10^6 g mol^{-1})	UHMWPE (3.2 x 10^6 g mol^{-1})
Filler	-	Graphene oxide; oxidation: Sun and Fujetsu method	-	Reduced graphene oxide; oxidation: Hummers method, reduction: ammonia and vitamin C	-	Graphene	Graphene
Filler size	-	D10, D50, D90 of 0.20 µm, 0.38 µm, 1.19 µm (source graphite >300 µm)	-	~45 µm (source graphite)	-	<5 µm	<7 µm
Filler content (wt.%)	-	1	-	1	-	1	1
Filler interlayer spacing (nm)	-	0.80	-	0.89 for GO, loss of ordering for RGO	-	-	-
Composite fabrication method	See (a)	See (b)	See (c)	See (d)	See (e)	See (f)	See (f)
Temperature (˚C)	205	205	180	180	230	230	230
Composite C/O (at./at.)	98.0 (SEM/EDS) 64.6 (XPS)	40.3 (SEM/EDS) 22.0 (XPS)	-	-	-	-	-
Surface roughness (nm)	37.14	32.16	-	-	-	-	-
Contact angle (˚)	93.50±0.30	94.93±0.98	-	-	93.4	86.5	86.5
X_c *via* XRD (%)	54.27	62.43	-	-	-	-	-
X_c *via* DSC (%)	57.05	64.95	34.72	39.26	56.11	66.99	67.11
Onset decomposition temperature (˚C)	423 (N_2)	428 (N_2)	-	-	413 (air)	427 (air)	429 (air)
Thermal decomposition mass residue (%)	0.30	0.00	-	-	-	-	-
Maximum force (N)	4450.625	4476.094	-	-	-	-	-
Crack initiation energy (J)	7869.4240	7164.7457	-	-	-	-	-
Crack propagation energy (J)	801.5489	497.0842	-	-	-	-	-
Yield point (N)	2695.625	2507.188	-	-	-	-	-
Compressive stiffness (N/m)	1092.663	1234.26127	-	-	-	-	-
Force at maximum displacement (N)	3211.719	4248.906	-	-	-	-	-
Maximum displacement (mm)	3.780	3.265	-	-	-	-	-
Microhardness (MPa)	-	-	5.86*	6.53*	480	630	600
Work of adhesion (J)	2.82×10^{-15}	6.17×10^{-15}	-	-	-	-	-
Surface potential gradient	None	Evidenced *via* EFM	-	-	-	-	-
Cell viability (%)	No toxicity	No toxicity	-	-	-	No toxicity	No toxicity

Reference	This work	This work	1	1	2	2	2

[(a)] UHMWPE powders were cold-pressed in stainless-steel cast at 10 MPa. Cast transferred into oven set at 205 ˚C and kept at 5 MPa. Cast removed and let cool down to RT under 10 MPa. (patent number BR10202400456)

[(b)] Mixture of GO and UHMWPE powders were cold-pressed in stainless-steel cast at 10 MPa. Cast transferred into oven set at 205 ˚C and kept at 5 MPa. Cast removed and let cool down to RT under 10 MPa. (patent number BR10202400456)

[(c)] UHMWPE powder sonication in ethanol followed by and drying. Molding of combined powders by hot pressing at 180 ˚C and 10 MPa for 30 min.

[(d)] RGO sonication in ethanol followed by addition of UHMWPE powder, sonication, and drying. Molding of combined powders by hot pressing at 180 ˚C and 10 MPa for 30 min.

[(e)] Dry mixing UHMWPE powders followed by 5-cylinder octa-screw kneading extrusion to yield strips that were cut into powder. Vacuum hot compression molding of rendered powders: hot pressing 230 ˚C for 5 min followed by gradual increase in pressure from 0 to 40 MPa in 10 min and maintained for 5 min.

[(f)] Dry mixing UHMWPE and graphene powders followed by 5-cylinder octa-screw kneading extrusion to yield strips that were cut into powder. Vacuum hot compression molding of rendered powders: hot pressing 230 ˚C for 5 min followed by gradual increase in pressure from 0 to 40 MPa in 10 min and maintained for 5 min.

* HV0.025 (Vickers microhardness scale using a load of 25 grams)

References

(1) Mindivan, F.; Çolak, A. Tribo-Material Based on a UHMWPE/RGOC Biocomposite for Using in Artificial Joints. *J Appl Polym Sci* **2021**, *138* (31). https://doi.org/10.1002/app.50768.

(2) Liu, C. Y.; Ishigami, A.; Kurose, T.; Ito, H. Wear Resistance of Graphene Reinforced Ultra-High Molecular Weight Polyethylene Nanocomposites Prepared by Octa-Screw Extrusion Process. *Compos B Eng* **2021**, *215*. https://doi.org/10.1016/j.compositesb.2021.108810.
